# Multiple action potentials and complex calcium dynamics following electroporation of S-HEK cells depend on gap junction coupling

**DOI:** 10.1038/s41598-026-63712-8

**Published:** 2026-07-28

**Authors:** Tina Batista Napotnik, Anja Blažič, Vid Jan, Klara Bulc Rozman, Rok Šmerc, Lea Rems

**Affiliations:** Faculty of Electrical Engineering, University of Ljubljana, Tržaška Cesta 25, 1000 Ljubljana, Slovenia

**Keywords:** Electroporation, Excitable cells, Action potential, Gap junctions, Intracellular calcium, Biological techniques, Biophysics, Cardiology, Cell biology

## Abstract

In recent years, irreversible electroporation has been increasingly used to treat cardiac arrhythmias, an approach termed pulsed field ablation. The mechanisms of how electroporation affects the generation and propagation of action potentials (AP) in the myocardium (and other excitable tissues) remain to be elucidated. In our previous in vitro studies using genetically engineered S-HEK cells as a simple model of excitable cells we identified two unexpected phenomena that remain unexplained: (1) multiple APs triggered by a single electric pulse, and (2) a pulse-induced complex (bi- or multiphasic) calcium response not observed in their non-excitable counterparts NS-HEK cells. In this study, we employed fluorescence microscopy for optical monitoring of APs and intracellular calcium, pharmacological interventions targeting specific ion channels, and molecular biology techniques to characterize the expression of selected ion channels in S-HEK and NS-HEK cells. Our findings show that (1) multiple APs result primarily from sustained AP propagation along the cell monolayer through gap junctions, and (2) the complex calcium response in S-HEK cells reflects synchronized calcium influx during these propagating APs, with extracellular calcium being primary source rather than internal store release.

**Supplementary Information:**

The online version contains supplementary material available at https://doi.org/10.1038/s41598-026-63712-8.

## Introduction

Electroporation is the phenomenon of increased cell membrane permeability induced by high-intensity pulsed electric fields. It has become a cornerstone of numerous biomedical applications including electrochemotherapy, gene electrotransfer, and nonthermal tumour ablation based on irreversible electroporation^1–4^. Most recently, irreversible electroporation has been adapted for cardiac arrhythmia treatment, where it is known as pulsed field ablation^5–7^. Within the past five years, this technology has rapidly advanced from the first in-human trials to clinical practice and represents one of the most significant recent innovations in interventional electrophysiology^8,9^. A critical aspect that remains to be fully understood is how electroporation affects the generation and propagation of action potentials (APs) in the myocardium, as evidenced by altered intracardiac electrograms recorded locally around the target ablation site^10,11^. Better understanding of the mechanisms underlying these alterations could lead to the development of periprocedural guidance that would inform clinicians about the likelihood of successful ablation and creation of permanent transmural lesions^12,13^. However, the diverse ion channel expression profile and heterogeneous structure of the myocardial tissue make it challenging to delineate the precise mechanisms by which electroporation affects cardiomyocyte excitability and AP propagation.

Simplified models of excitable cells developed through genetic engineering have emerged as valuable platforms for studying the fundamental aspects of cellular excitability and its modulating factors^14–17^. One such example are human embryonic kidney (HEK) cells expressing voltage-gated sodium channels and inward rectifying potassium channels—a minimal complement of ion channels necessary for electrical excitability^15^. A key feature of these spiking HEK (S-HEK) cell systems is their ability to form electrically coupled syncytial networks via endogenous gap junctions (predominantly connexins 43 and 45), similar to those expressed in cardiomyocytes. When grown as confluent monolayers, S-HEK cells support intercellular electrical communication and coordinated propagation of APs, mimicking the collective behaviour of natural excitable tissues such as the myocardium. Studies with S-HEK cells have offered important insights into geometry-dependent arrhythmias^18^, formation of bioelectric domain walls in homogeneous tissues^19^, and generation of AP at the interface of two unexcitable tissues^20^.

In our previous studies^21–24^ we utilized S-HEK monolayers to investigate the effects of electroporation on AP generation. When exposing these monolayers to pulses of increasing electric field strength, we observed that mild electroporation (without detectable propidium uptake) prolongs APs due to additional leak current across the permeabilized membrane and leads to sustained membrane depolarization. We also identified two unexpected phenomena that remained unexplained. At low to intermediate electric field strengths (e.g., for a single 100 µs pulse, 126–200 V/cm)^21,22^, S-HEK cells within the monitored field of view triggered either a single or multiple APs in response to a single electric pulse. Furthermore, S-HEK cells exhibited a complex calcium response not observed in their non-excitable counterparts (NS-HEK cells expressing only Na_V_1.5 channels). This calcium response featured two or more distinct peaks, with the second peak typically larger than the first. Both phenomena were consistently observed in response to electric pulses across a wide pulse duration range, from 500 to 1 ms^22^.

The triggering of multiple APs could result from propagating waves initiated by the applied pulse^14,16^. Optical mapping studies of S-HEK monolayers have revealed that AP waves emanating from local sources can establish spontaneously, enabling these cells to become “spontaneously spiking”^14,16^. Alternatively, additional APs (afterdepolarizations or oscillatory behavior) have been observed in experiments on isolated ventricular myocytes^25,26^ and were attributed to the interplay between electroporation leak currents and activation of L-type calcium channels^25^. Since some voltage-gated calcium channels are endogenously expressed in HEK cells^27^, further investigation is required to determine the precise mechanism underlying the multiple APs response.

The complex calcium response in S-HEK cells suggests a distinctive interplay between membrane excitability and calcium handling mechanisms. The initial calcium peak likely results from membrane electroporation and/or opening of the endogenous voltage-gated or other calcium channels.^21,22^ However, the second, typically larger calcium peak may involve active amplification through more complex calcium signalling pathways, such as calcium release from internal stores (endoplasmic reticulum, ER) via ryanodine or inositol 1,4,5-trisphosphate (IP3) receptors through calcium-induced calcium release (CICR)^28^, store-operated calcium entry^29^, intercellular calcium waves^30^, or depolarization-dependent influx of Ca^2+^ through voltage-gated calcium channels^31^.

In this study, we aimed to elucidate: (1) ionic mechanisms responsible for multiple AP generation in S-HEK cells following a single electric pulse, and (2) the source of the complex (bi- or multiphasic) calcium response observed exclusively in S-HEK cells. We employed a combination of experimental approaches including fluorescence microscopy for optical monitoring of APs and intracellular calcium, pharmacological interventions targeting specific ion channels, and molecular biology techniques to characterize the expression of selected ion channels in S-HEK and NS-HEK cells. Our findings show that (1) multiple APs, whether spontaneous or pulse-stimulated, mainly result from AP propagation, and (2) the complex calcium response to electric pulses in S-HEK cells is synchronized with triggering of APs after electroporation and thus not observed in NS-HEK cells. These results contribute to clarification of basic mechanisms of electroporation of excitable cells and thus to development of electroporation-based treatments of excitable tissues.

## Results

### Pharmacological characterization of spontaneous action potentials in S-HEK cells

S-HEK monolayers with high cell density often exhibit spontaneous APs (spontaneously spiking cultures), even before any pulse is delivered. We hypothesized that spontaneous APs and the multiple APs triggered by a single electric pulse share the same underlying mechanism: AP propagation across the cell monolayer in spatio-temporal patterns (e.g., spirals), as reported in other studies^14–16^. As these propagating waves repeatedly cross a given region, multiple APs are detected there over the observation period.

S-HEK cells were grown in a monolayer within an imaging chamber, stained with the fluorescent potentiometric dye ElectroFluor630, incubated in low K^+^ Tyrode Buffer, and observed under a fluorescence microscope at room temperature, as in our preceding studies^21–24^. We monitored transmembrane voltage (TMV) signal combined from all the cells in a field of view. Cells in a monolayer function as a syncytium and are synchronized due to gap junction connections. We confirmed this by separately analyzing the TMV signals from four quadrants of the captured images; the shape and timing of these signals were indeed the same as the signal from the whole field of view (Suppl. Fig. S1).

To investigate the underlying mechanism of spontaneous APs, we first examined whether we could inhibit spontaneous spiking using three pharmacological inhibitors targeting different ion channels and intercellular communication pathways: potassium channel inhibitor tetraethylammonium (TEA), calcium channel inhibitor verapamil, and gap junction inhibitor carbenoxolone (CBX). The inhibitor concentrations and monitoring times were determined based on the literature^32–35^ and our own observations. At the concentrations used, verapamil and TEA act practically instantly^32,36^, while CBX requires 10–30 min^34,35^.

**Fig. 1:**
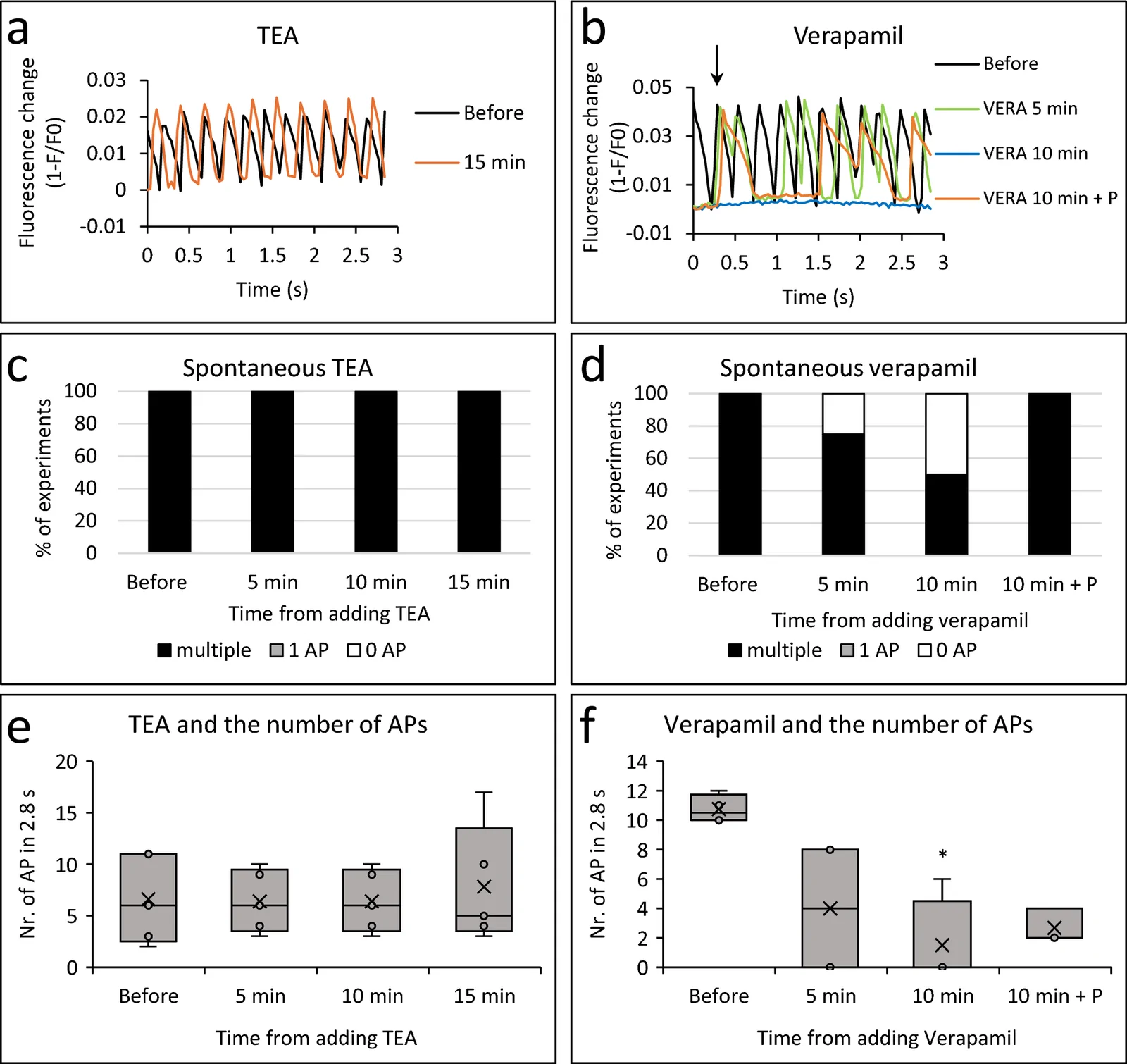
The effect of TEA and verapamil on spontaneous APs in S-HEK monolayers. (**a, b**) Transmembrane voltage (TMV) responses before and after adding 50 mM TEA (**a**) or 25 µM verapamil (**b**) from a representative experiment. Presented curves show a fluorescent potentiometric dye ElectroFluor630 fluorescence signal combined from the membranes of all the cells in the field of view and corrected for photobleaching. When spontaneous APs had terminated after adding verapamil, a single 100 µs, 176 V/cm electric pulse was applied to test whether APs could still be triggered; this corresponds to the “10 min + P” trace in (**b**). Arrow marks the pulse delivery. (**c**, **d**) Types of TMV responses (0 AP, 1 AP or multiple APs) after adding TEA (**c**) or verapamil (**d**), expressed in % of all experiments. (**e**, **f**) The effects of TEA (**e**) or verapamil (**f**) on the number of APs during image acquisition (around 2.8 s), shown in box-whiskers plots. Number of experiments: 5 (panels c, e); 4 (panels d, f). *—statistical difference between values at different time points and before adding a compound, Kruskal–Wallis One Way Analysis of Variance on Ranks followed by Dunn’s test for multiple comparisons versus before adding a compound, p < 0.05.

Tetraethylammonium (TEA) is an inhibitor of various potassium channels (K_V_1-3, K_Ca_1, 2, TRP cation channels), but not K_ir_ channels that are expressed in genetically modified S-HEK cells^33,37,38^. Application of 50 mM TEA did not affect the rate of spontaneous APs, the number of APs during image acquisition (around 2.8 s) remained stable for 15 min after adding TEA to cell cultures (Fig. 1a, c, e).

Verapamil is primarily an inhibitor of voltage-gated calcium channels (L- and T-type)^32^. although it can also affect other channels including K_ir_^39^, but not connexins Cx43^40^. In contrast to TEA, 25 µM verapamil caused a nonsignificant decrease in rate of spontaneous APs (Fig. 1b, d, f, Fischer exact test). The number of APs during image acquisition decreased significantly over 10 min after adding verapamil to cell cultures (Fig. 1f). In approximately 50% of cases, spontaneous APs ceased entirely. To test whether multiple APs could still be triggered in samples where spontaneous APs had ceased, we applied a single electric pulse of 100 µs, 176 V/cm, 10 min after adding verapamil. In all cases, this pulse still triggered multiple APs (Fig. 1f).

**Fig. 2:**
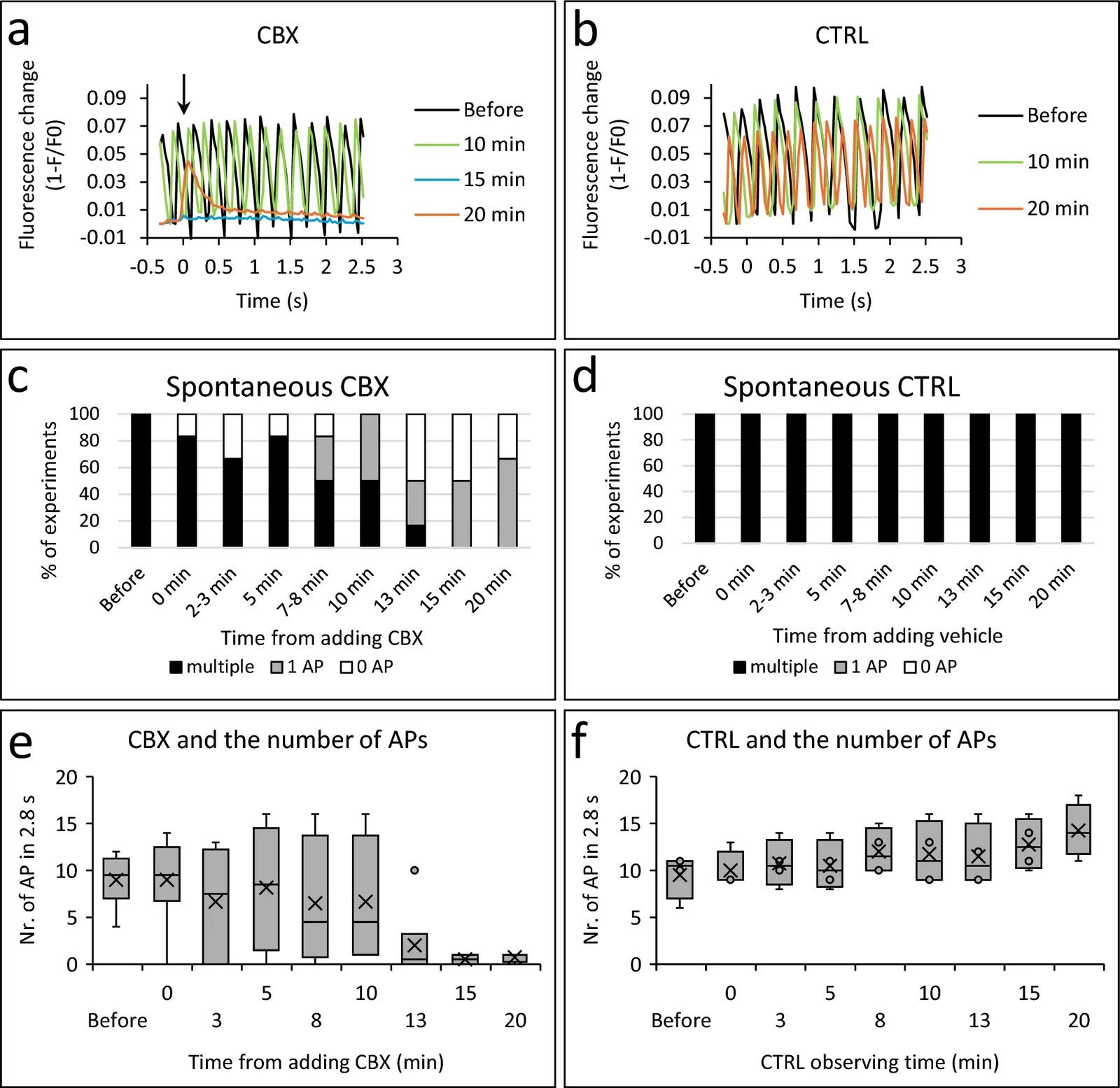
The effect of carbenoxolone (CBX) on spontaneous APs in S-HEK cells. **a**, **b** Transmembrane voltage (TMV) responses before and after adding 100 µM CBX (**a**) or vehicle (0.4% ethanol) for control (CTRL) (**b**) from a representative experiment. Presented curves show fluorescent potentiometric dye ElectroFluor630 fluorescence signal combined from the membranes of all the cells in the field of view and corrected for photobleaching. After the spontaneous APs had terminated, a single 100 µs electric pulse (126–200 V/cm) was applied to test whether APs could still be triggered; this corresponds to the “20 min” trace in (**a**). Arrow marks the pulse delivery. **c**, **d** Types of TMV responses (0 AP, 1 AP or multiple APs) after adding CBX (**c**) or vehicle for CTRL (**d**), expressed in % of all experiments. **e**, **f** The effects of CBX (**e**) or vehicle for CTRL (**f**) on the number of APs during image acquisition (around 2.8 s), shown in box-whiskers plots. Number of experiments: 6 (panels **c**, **e**); 4 (panels **d**, **f**).

In contrast, the gap junction inhibitor carbenoxolone (CBX, 100 µM)^34^ had a more pronounced effect (Fig. 2). After adding CBX to cells with spontaneous APs, we observed that spontaneous APs stopped around 13 min (Fig. 2a). The occurrence of spontaneous APs was indeed significantly different from their occurrence before adding CBX from 13th min up to 20th min, as well as from the corresponding responses in control experiments (Fisher’s exact test, p < 0.05). In each experiment after termination of spontaneous APs, we applied a single 100 µs electric pulse to test whether APs could still be triggered with pulses (electrostimulation). The applied electric field strength E (126–200 V/cm) was chosen from the data from previous publications^21,22^ as most likely to trigger multiple APs and adjusted to each experiment to get a response. At 15 min after adding CBX to cells we were no longer able to trigger multiple APs, observing only a single AP or no response (Fig. 2c, e). Addition of vehicle alone (0.4% ethanol, the same amount as in CBX experiments) in control experiments did not cause termination of spontaneous APs for at least 20 min (Fig. 2b, d, f).

### Multiple action potentials after single electric pulse application in S-HEK cells are dependent on gap junctions and action potential propagation

Having observed that CBX terminated spontaneous APs in S-HEK monolayers, we next tested whether CBX also affects multiple APs triggered by electric pulse. If so, this would support our first hypothesis that both spontaneous and pulse-triggered multiple APs arise from the same propagation mechanism.

For these experiments, we used S-HEK samples that did not exhibit spontaneous APs, as confirmed in recordings at the start of each experiment. To minimize electroporation, cells were stimulated at the lowest pulse amplitude that triggered APs (the threshold amplitude). Because this threshold varies slightly between experiments^21,22^, it was determined separately for each one: cells were monitored between the electrodes (the pulse-exposed area) and exposed single 100 µs electric pulses of increasing amplitude (E = 126–200 V/cm) every 2 min until a response was triggered. Based on the type of response obtained, the samples were assigned to two groups: (1) samples that responded with multiple APs and (2) samples that responded with only a single AP between the electrodes. After group assignment, 100 µM CBX was added, and the same E that had initially triggered the response was reapplied at 0 min, 2–3 min, 5 min, 7–8 min, 10 min, 15 min and 20 min after adding CBX, to monitor changes in TMV response.

**Fig. 3:**
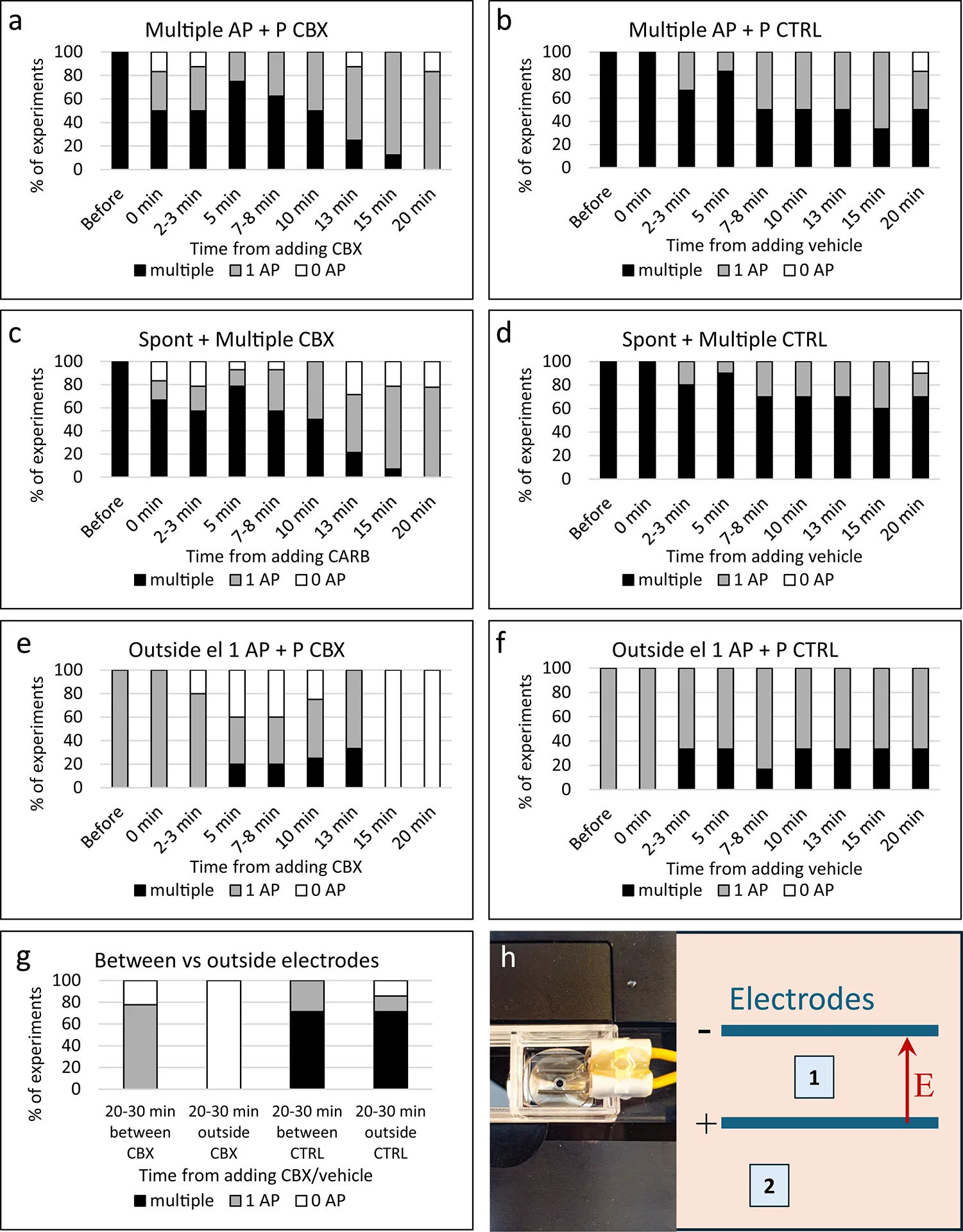
The effect of carbenoxolone (CBX) on multiple APs and AP propagation in S-HEK monolayers. **a**–**f** Types of TMV responses (0 AP, 1 AP or multiple APs) over time after adding 100 µM CBX (**a**, **c**, **e**) or vehicle for control (CTRL) (**b**, **d**, **f**), expressed in % of all experiments. **a**, **b** Responses recorded between the electrodes in cultures that showed no spontaneous APs but produced multiple APs in response to a single 100 µs pulse (126–200 V/cm). **c**, **d** Pooled results combining spontaneous (Fig. 2a, b) and pulse-triggered multiple APs (Fig. 3a, b). **e**, **f** TMV responses recorded outside of the electrodes in experiments where a pulse triggered a single AP between the electrodes. Number of experiments (N): **a**: 8, **b**: 6, **c**: 14, **d**: 10, **e**: 5, **f**: 6. **g** Comparison of the TMV responses recorded between the electrodes versus outside them, 20–30 min after adding CBX or vehicle (control); N: 9 for CBX and 7 for CTRL. **h** Photograph and schematic of the imaging chamber showing the two observation areas: (1) between the electrodes (i.e., the area with pulse exposure), and (2) outside the electrodes (i.e., area without pulse exposure). The schematic is not drawn to scale.

To determine whether CBX inhibits multiple APs, we continued to monitor the first group between the electrodes (area 1 in Fig. 3h) as CBX was added. Indeed, multiple APs typically ceased around 13 min later; thereafter, the cells responded with only a single AP or no response (Fig. 3a). From the 13th to the 20th min, the occurrence of multiple APs differed significantly from that before adding CBX (Fisher’s exact test, p < 0.05, applied throughout Fig. 3a–d). This timing of CBX inhibition was similar to that observed in the spontaneously spiking samples (Fig. 2). In control experiments (vehicle only), the occurrence of multiple APs after pulsing declined somewhat over time, though it was not significantly different from before adding the vehicle (Fig. 3b). Because of this decline in the controls, the CBX-treated and control samples did not differ significantly from each other at any time point.

During the experiments, we observed that cell responses could change dynamically: in several cases a pulse triggered APs that then persisted spontaneously for several minutes, while in others initially spontaneous APs ceased and the cells had to be pulsed to trigger APs. Because of this dynamic behavior and overlap between spontaneous and pulse-triggered responses, we pooled the data from experiments with both spontaneously spiking samples (Fig. 2a) and samples with pulse-triggered multiple APs (Fig. 3a). In this pooled analysis (Fig. 3c), the occurrence of multiple APs (whether spontaneous or pulse-triggered) differed significantly from that before adding CBX from as early as the 3rd minute. In control experiments (with vehicle only), the occurrence of multiple APs was not significantly different from before adding the vehicle (Fig. 3d), but it differed significantly from the CBX-treated experiments from the 13th minute onward. Similar results were obtained when using 10 µs pulses to trigger APs (Suppl. Fig. S2).

To test directly whether CBX blocks AP propagation, we examined the second group. In these experiments, the samples were monitored outside the electrodes (area 2 in Fig. 3h), reached by moving the microscope stage. Because this area receives no pulse, any AP detected there must have propagated across the monolayer from the stimulated region. Before adding CBX, such propagated APs were present; from 15 min after adding CBX, they ceased to appear (Fig. 3e). The occurrence of any AP was significantly different from their occurrence before adding CBX from 15th min up to 20th min (Fisher’s exact test, p < 0.05). In contrast, in control experiments (vehicle only), APs continued to appear for at least 20 min (Fig. 3f). Occasionally, samples that initially triggered a single AP produced multiple APs in response to subsequent pulses (Fig. 3e, f).

In all the experiments with CBX, 20–30 min after adding CBX or vehicle, we also monitored TMV both between the electrodes and, by moving the stage, immediately afterward outside them, or vice versa (Fig. 3g). In every experiment, CBX both prevented multiple APs between the electrodes and blocked any AP outside them, indicating that these multiple APs are generated by propagation through gap junctions.

Throughout these experiments, the TMV signal reflects the activity of all cells in the field of view collectively. Any change we observed could therefore, in principle, reflect asynchronous or spatially fragmented firing among individual cells rather than a genuine change in their activity. To exclude this, we applied the four-quadrant analysis to randomly selected CBX experiments; in all tested five, the quadrant signals again matched the whole-field trace (Suppl. Fig. S3), confirming that firing remained synchronized across the field. Asynchronous firing is therefore highly unlikely to account for the changes we observed.

**Fig. 4:**
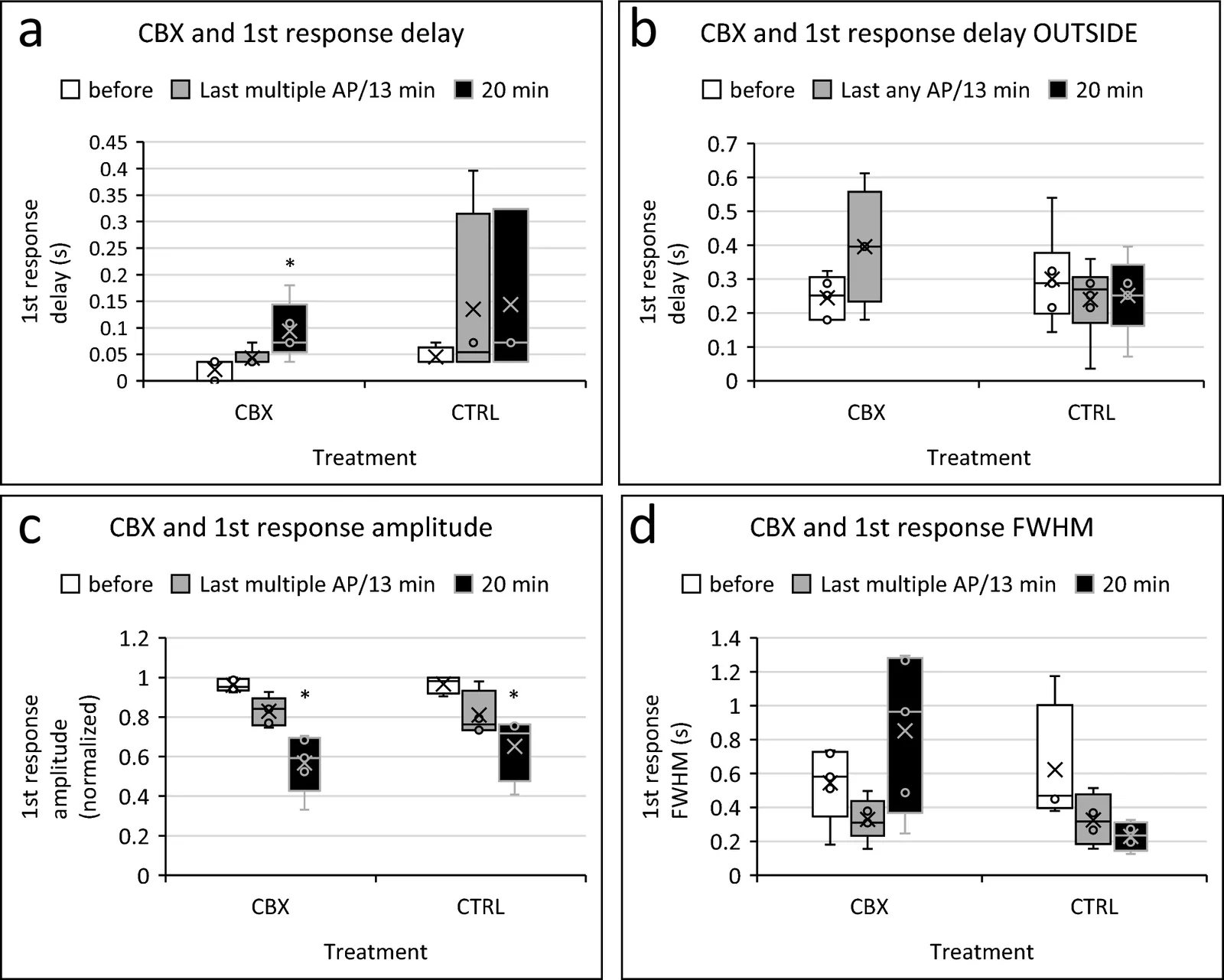
Characteristics of the first AP recorded in response to a single 100 µs electric pulse (126–200 V/cm) in S-HEK monolayers, analyzed from the responses in Fig. 3. Characteristics were determined at different times following carbenoxolone (CBX) or vehicle (control, CTRL). **a**, **b** Delay between pulse onset and the first AP peak, for cells recorded between the electrodes (**a**) and outside the electrodes (**b**). **c** Normalized amplitude and **d** full width at half maximum (FWHM) of the first AP. Number of experiments: a, c, d: 5 for CBX and 4 for CTRL; b: 5 for CBX and 6 for CTRL (some of the results from Fig. 3 were not eligible for this analysis due to the lack of APs or spontaneous APs at the analysed points). Box-and-whisker plots; *—statistical difference between values at different time points, ANOVA On Ranks followed by Tukey test for all pairwise multiple comparisons, p < 0.05.

We additionally analyzed AP characteristics in the pulse-triggered multiple-AP responses (Fig. 3a, b), by comparing CBX-treated with control samples at three matched time points: in CBX-treated samples, before adding CBX, at the time of the last observed multiple-AP response (around 13 min), and at 20 min; in control samples, before adding vehicle and at 13 and 20 min afterward (Fig. 4). For the first AP of each response, we examined the delay between pulse onset and the AP peak value, the amplitude, and the full-width half-maximum (FWHM). While differences between control and CBX-treated samples were not statistically significant, they showed trends consistent with CBX blocking gap junctions and AP propagation. The pulse-to-AP delay tended to be shorter in CBX-treated samples than in controls (Fig. 4a). This is consistent with inhibited AP propagation, as APs no longer propagate from the area near the electrodes (where the local electric field is highest) to the observation area but must instead be triggered directly in the observed cells. Supporting this interpretation, the opposite trend was seen when observing cells outside the electrodes (Fig. 3e, f); the last recorded AP before propagation ceased was more delayed compared to the AP recorded before CBX addition (Fig. 4b), confirming the progressive inhibition of AP propagation by CBX. CBX had no effect on AP amplitude compared to controls—both showed similar decreases due to fluorescence fading (Fig. 4c). Interestingly, CBX tended to prolong AP FWHM (Fig. 4d), an effect that could reflect its action on ion channels other than gap junctions.

### Confirmation of ion channels expression at the molecular level

Our experiments with CBX indicated that spontaneous APs are mainly governed by AP propagation through gap junctions. Furthermore, experiments with verapamil suggested that endogenous voltage-gated calcium channels may also influence AP propagation in spontaneously spiking S-HEK cells. To test the hypothesis that connexins, which form gap junctions, and voltage-gated calcium channels are indeed expressed in these cells at the molecular level, we employed immunofluorescence and Western blot analysis.

According to the Human Protein Atlas^41^ (see Suppl. Table S2) and preceding studies^14,42,43^, HEK293 cells predominantly express gap junction connexins Cx43 and Cx45 and voltage-gated T-type calcium channels Ca_V_3.2. Thus, we tested the expression of these proteins in both S-HEK and NS-HEK cells. In addition, since these cells constitutively express voltage gated Na_V_1.5 channels and conditionally express K_ir_2.1 channels, we tested the expression of these proteins as well.

**Fig. 5:**
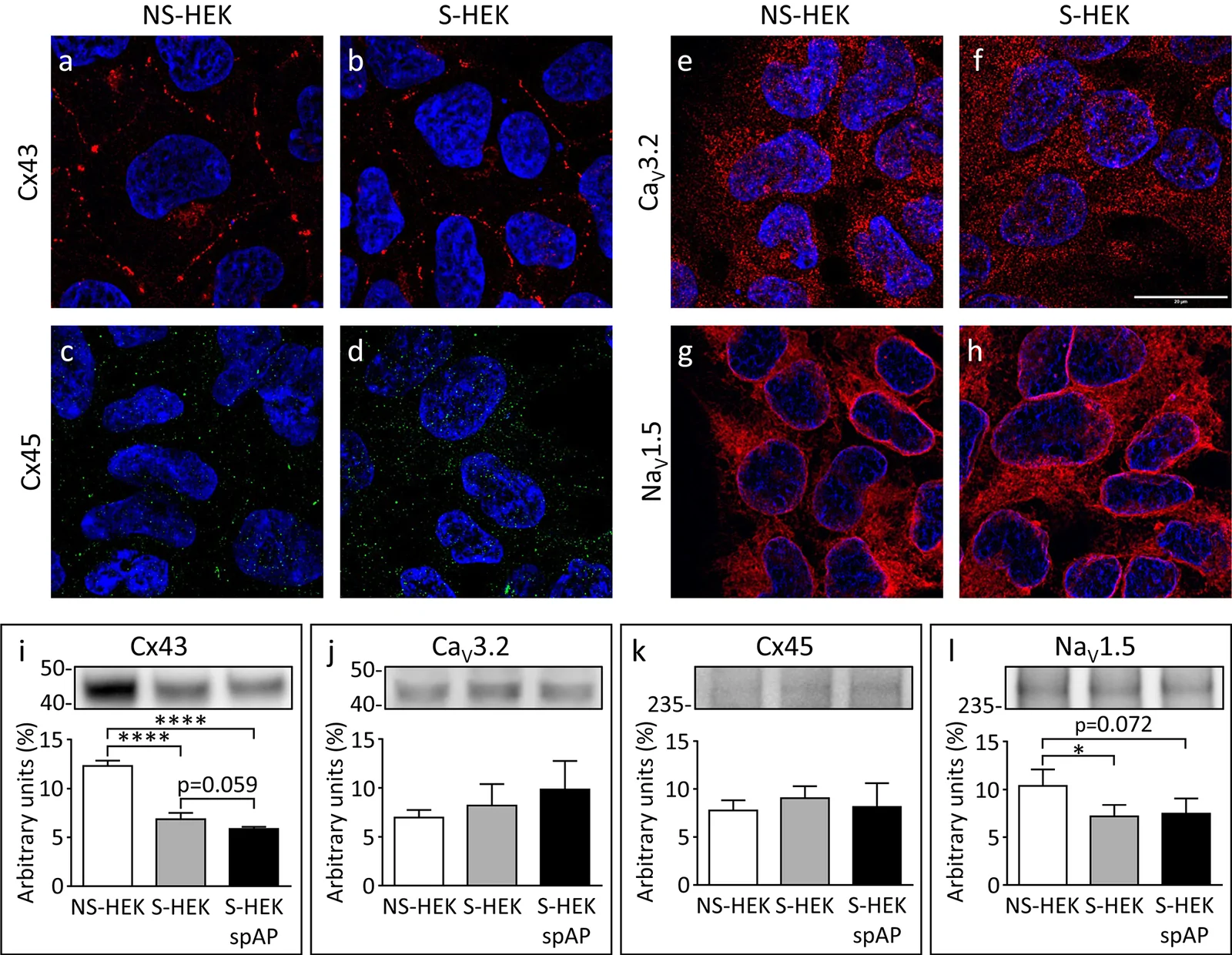
Protein expression of ion channels and gap junction components in excitable S-HEK and non-excitable NS-HEK cells. **a**–**h** Localization of Cx43 (red; a, b), Cx45 (green; c, d), Ca_V_3.2 (red; e, f) and Na_V_1.5 (red; g, h) by immunofluorescence in NS-HEK and S-HEK. Nuclei are labeled in blue. Number of experiments: 3. Scale bar, 20 µm. **i**–**l** Immunoblotting was used to estimate protein abundance of connexin 43 (Cx43; i), voltage gated T-type calcium channel 3.2 (Ca_V_3.2; j), connexin 45 (Cx45; k), and voltage-gated sodium channels (Na_V_1.5; l) in non-excitable (NS-HEK), excitable without spontaneous APs (S-HEK), and excitable HEK cells with spontaneous APs (S-HEK spAP). Results are presented as means ± SD. *statistical difference between groups (One-way ANOVA with Bonferroni’s post-hoc analysis, p < 0.05). Number of experiments: 4.

Using immunofluorescence, we confirmed that S-HEK and NS-HEK cells express gap junction connexins Cx43 and Cx45, Ca_V_3.2 and Na_V_1.5 channels (Fig. 5a–h). To quantify protein expression, we performed Western blot analysis (Fig. 5i–l). We investigated whether there were any differences in the expression of specific ion channels and gap junction components between non-excitable HEK cells (NS-HEK), excitable HEK cells that did not exhibit spontaneous APs (S-HEK), and excitable HEK cells that exhibited spontaneous APs (S-HEK spAP). Interestingly, the expression of Cx43 and Na_V_1.5 was significantly lower in S-HEK cells compared to NS-HEK cells (Fig. 5i, l). Other differences in expression levels were not statistically significant.

We also aimed to evaluate protein abundance of heterologous K_ir_2.1 in NS-HEK and S-HEK cells to confirm the presence of K_ir_2.1 only in S-HEK cells (our cells have a tet-on system for doxycycline-inducible gene expression of murine K_ir_2.1). However, attempts to confirm K_ir_2.1 expression using two commercial antibodies were unsuccessful due to insufficient antibody specificity for our cells (Suppl. Fig. S4-6). Since K_ir_2.1 is tagged with cyan fluorescent protein, we previously confirmed its expression by visualizing cyan fluorescence in S-HEK cells but not in NS-HEK cells^21^.

### The calcium peaks in S-HEK and NS-HEK depend on extracellular calcium

**Fig. 6:**
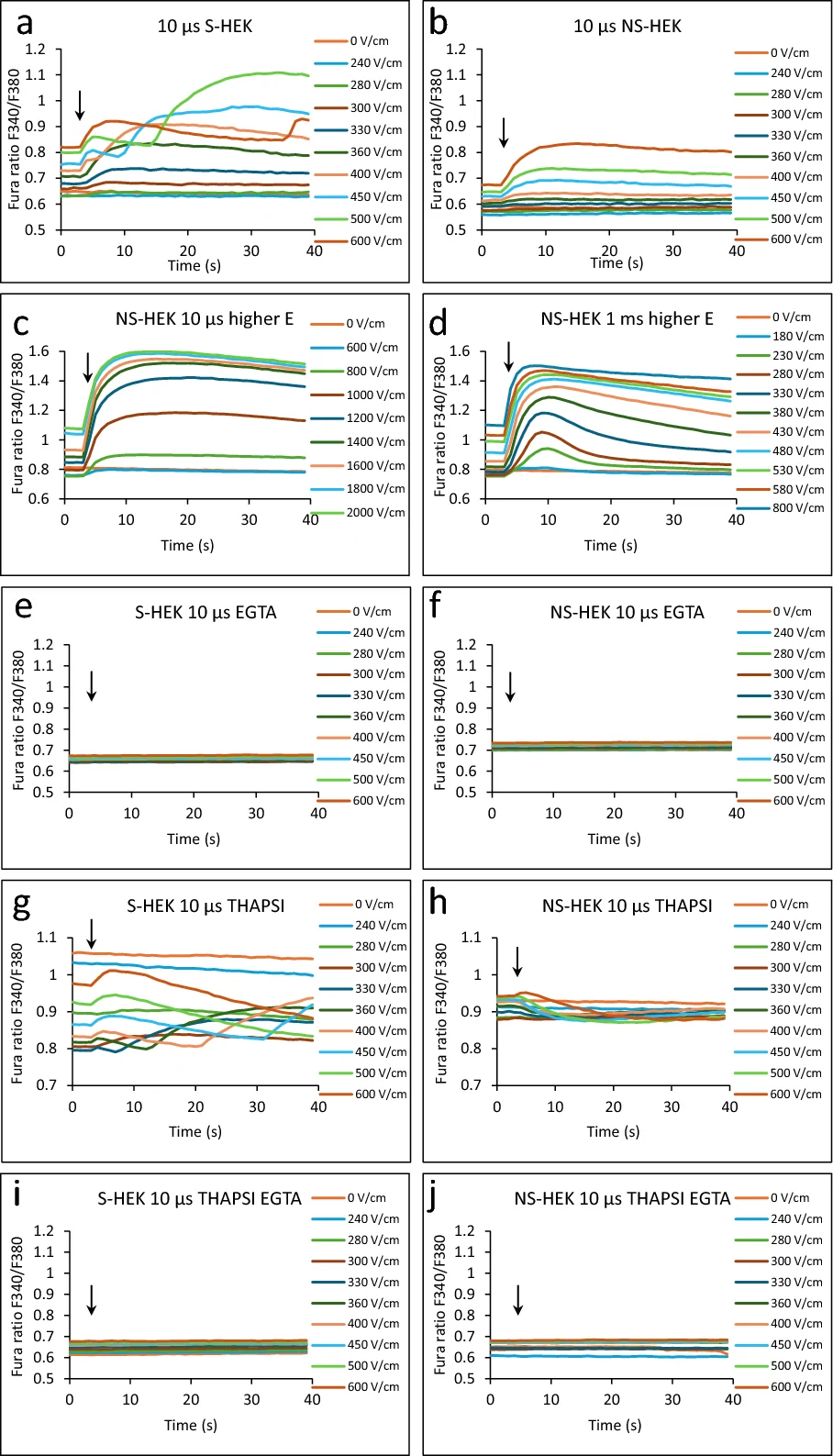
Ca^2⁺^ responses of S-HEK and NS-HEK monolayers to single electric pulses of increasing amplitude (i.e., increasing electric field strength) delivered 2 min apart, recorded at different experimental conditions. Responses were determined by Fura-2 ratio 340/380, averaged over the whole image. Each panel shows a representative experiment. A pulse was delivered at around 4^th^ second after the image acquisition started (arrows). **a**–**d** Responses obtained in our standard low K^+^ Tyrode buffer. **a**: S-HEK cells, 10 µs pulse. **b**: NS-HEK cells, 10 µs pulse. **c**: NS-HEK cells, 10 µs pulse of higher electric field than in panel b. **d**: NS-HEK cells, 1 ms pulse, higher electric field than in panel b. Data for both a and b are from our preceding study^22^. Number of experiments: a: 8, b: 7, c: 7, d: 8. **e**–**j** Responses obtained after manipulation of extracellular and intracellular Ca^2⁺^ sources. **e**: S-HEK cells, 10 µs pulse, 5 µM EGTA in extracellular medium without Ca^2+^. **f**: NS-HEK cells, same conditions as in panel e. **g**: S-HEK cells, 10 µs pulse, 30 min incubation with 200 nM thapsigargin to deplete internal Ca^2+^ stores. **h**: NS-HEK cells, same conditions as in panel g. **i**: S-HEK cells, 10 µs pulse, 5 µM EGTA in extracellular medium without Ca^2+^ and thapsigargin to deplete internal Ca^2+^ stores. **j**: NS-HEK cells, same conditions as in panel i. Number of experiments: e: 3, f: 3, g: 5, h: 3, i: 3, j: 3.

In our previous reports^21,22^, we exposed S-HEK and NS-HEK cells to a series of single electric pulses delivered 2 min apart, increasing the amplitude progressively from one pulse to the next. At intermediate and higher pulse amplitudes, we observed a complex Ca^2+^ response in S-HEK cells but not in NS-HEK cells (see Fig. 6a, b). In S-HEK cells, the initial pulse-triggered peak was followed by a higher, amplified peak (sometimes even more peaks) a few seconds or a few tens of seconds later. In contrast, NS-HEK cells always responded with only one Ca^2+^ peak and a complex response was never observed after any of the tested pulses. Here, we re-examined this response using the same protocol and the ratiometric Ca^2+^ indicator Fura-2, analyzing the signal collectively across all cells in the field of view. At the single-cell level, the complex S-HEK response reflects two or more Ca^2⁺^ peaks in each cell, synchronized across the monitored cells (Suppl. Fig. S7). In contrast, NS-HEK cells show only a single peak per cell (Suppl. Fig. S8).

We hypothesized that the complex Ca^2+^ response in S-HEK cells is related to the excitability of S-HEK cells. Following this hypothesis, we investigated three aspects: (1) Can a complex Ca^2+^ response in NS-HEK cells be triggered with higher pulse amplitude? (2) What is the source of calcium in response to electric pulses in both cells? (3) How do gap junctions influence Ca^2+^ response in S-HEK and NS-HEK cells?

The Ca^2⁺^ response scaled with pulse amplitude and, at every amplitude, the response was smaller in NS-HEK than in S-HEK cells (Fig. 6a, b), as we reported previously^22^. We reasoned that NS-HEK might simply not have reached the threshold for a complex Ca^2+^ response, which in S-HEK cells emerged only at intermediate-to-high field strengths. To test this, we exposed NS-HEK cells to pulse amplitudes higher than those used previously. Since we observed the complex Ca^2+^ response in S-HEK with pulses of different durations (from 500 ns to 1 ms)^22^, we chose 10 µs and 1 ms pulses for this study (Fig. 6c, d). However, even pulses of higher amplitudes did not trigger a complex Ca^2+^ response in NS-HEK cells.

To elucidate the source of Ca^2+^ in the observed responses, we tested three conditions: (1) depletion of extracellular Ca^2+^ using 5 µM EGTA in Ca^2+^-free medium, (2) depletion of intracellular Ca^2+^ stores from the endoplasmic reticulum using 200 nM thapsigargin^44^, and (3) depletion of both extracellular and intracellular Ca^2+^ pools using both EGTA and thapsigargin.

In both S-HEK and NS-HEK cells, Ca^2+^ influx originated from the extracellular medium since no Ca^2+^ response was observed when EGTA was present in Ca^2+^-free medium (Fig. 6e, f, i, j). Moreover, when intracellular Ca^2+^ stores were depleted by thapsigargin, but with Ca^2+^ present in the extracellular medium, we still detected a Ca^2+^ response in both S-HEK and NS-HEK cells (Fig. 6g, h), a complex response in S-HEK and a monophasic response in NS-HEK. This demonstrates that additional peaks in S-HEK cells are unlikely to be due to Ca^2+^ release from intracellular stores. Cells that were not exposed to electric pulses did not exhibit a Ca^2+^ response under any of these conditions.

**Fig. 7:**
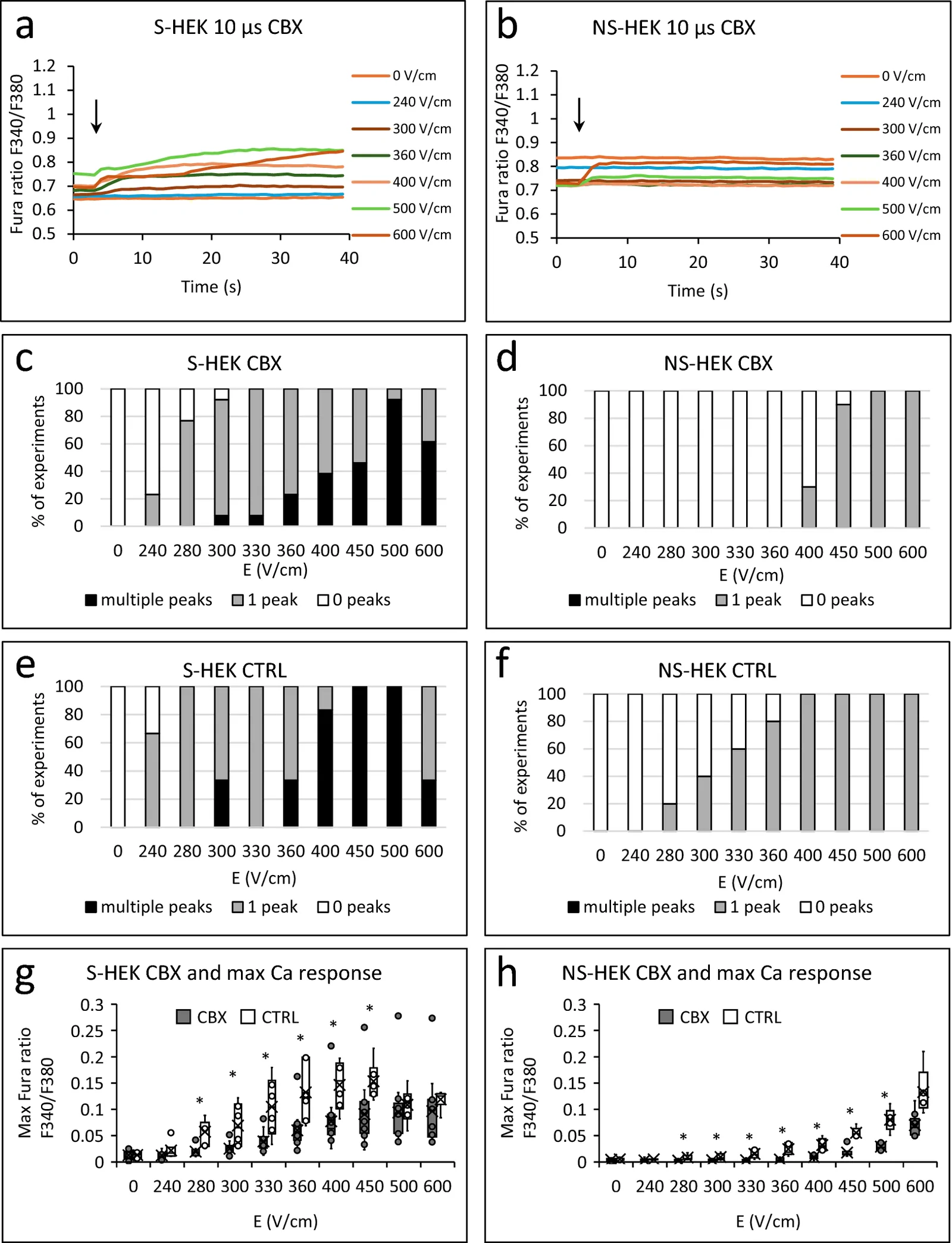
The effect of gap junction inhibitor carbenoxolone (CBX) on Ca^2+^ response to 10 µs electric pulses in S-HEK and NS-HEK cells. Cells were incubated in 100 µM CBX for 15 min and then exposed to 10 µs electric pulses of increasing electric field delivered 2 min apart as in Fig. 6. Ca^2+^ responses were determined by Fura-2 ratio 340/380, averaged over the whole image. A pulse was delivered at around 4th second after the image acquisition started (arrows). **a**, **b** Ca^2+^ responses from representative experiments in S-HEK (**a**) and NS-HEK (**b**). **c**–**f** Different types of Ca^2+^ responses (0 peak, 1 peak or multiple peaks) in S-HEK (**c**, **e**) and NS-HEK (**d**, **f**) after adding 100 µM CBX (**c**, **d**) or or vehicle for control (CTRL) (**e**, **f**). Number of experiments: c: 13, d: 10, e: 6, f: 5. **g**, **h** Maximum Ca^2⁺^ responses, determined after subtracting each signal’s baseline value at t = 0 s. Results are presented as box plots. Number of experiments: g: 13 for CBX and 6 for CTRL, h: 10 for CBX and 5 for CTRL. *—statistical difference between CBX and CTRL (Mann–Whitney Rank Sum Test, p < 0.05).

Gap junction inhibitor CBX notably attenuated the multiphasic response in S-HEK but did not abolish it completely (Fig. 7a, c, e, for control without CBX see Fig. 6a). The analysis of Ca^2+^ response at individual cell level revealed that CBX-treated S-HEK cells still exhibit multiple Ca^2+^ peaks (Suppl. Fig. S9), however, in less synchronized pattern than control cells (Suppl. Fig. S7). In CBX-treated NS-HEK, the Ca^2+^ response remains monophasic, with a single peak after pulse application (Fig. 7b, d, f; for control without CBX see Fig. 6b).

In S-HEK cells, CBX did not significantly change the threshold electric field for a Ca^2⁺^ response (Fig. 7c, e): the occurrence of Ca^2⁺^ peaks became significantly higher than in the no-pulse control from 280 V/cm both with and without CBX (Fisher’s exact test, p < 0.05). Nevertheless, responses were slightly less frequent with CBX. In NS-HEK cells, by contrast, CBX raised the threshold: the occurrence became significant at 450 V/cm with CBX versus 360 V/cm without CBX (Fig. 7d, f). In both cell types, the maximum Ca^2⁺^ response was lower with CBX than without CBX for most of the applied E (Fig. 7g, h, Mann–Whitney Rank Sum Test, p < 0.05).

We also noted that the baseline intracellular Ca^2⁺^ increased during incubation with CBX in NS-HEK cells, followed by a gradual decrease during the course of experiment (Fig. 7b, Suppl. Fig. S10). The increased basal Ca^2⁺^ level in cells exposed to CBX is consistent with other findings^45^. On the other hand, in CBX-treated S-HEK cells (Fig. 7a), Ca^2⁺^ baseline rose slowly throughout the experiment, similarly as in non-treated control cells (Suppl. Fig. S10), as we observed previously^21^, owing to our experimental conditions. To keep each experiment short, we applied pulses of increasing amplitude at 2 min intervals, which limited the duration of these experiments to 18 min. Nevertheless, the higher-amplitude pulses applied later in the sequence left the plasma membrane and Ca^2⁺^ baseline incompletely recovered between pulses, further raising the baseline.

### Simultaneous measurements of transmembrane voltage and internal Ca^2+^

**Fig. 8:**
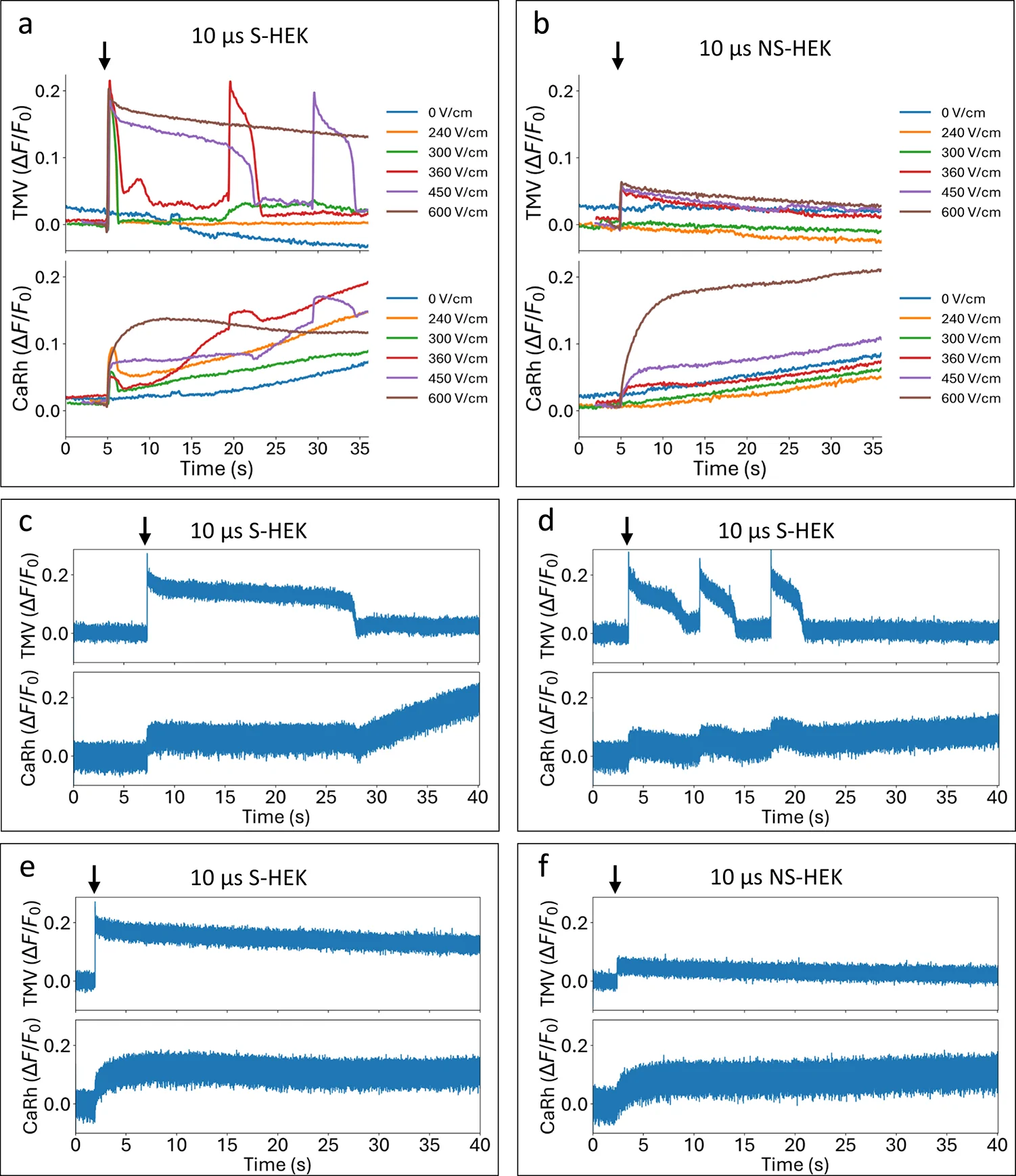
Simultaneous measurements of transmembrane voltage (TMV) and Ca^2⁺^ levels (CaRh) in S-HEK and NS-HEK cells. Ca^2^⁺ indicator Rhod-2/AM and voltage-sensitive indicator FluoVolt™ with Powerload™ were used. **a**–**b** TMV and Ca^2⁺^ in response to 10 μs pulses of increasing electric field strength from representative experiments. For each electric field increment we moved our field of view to another part of the coverslip to avoid the effects of photobleaching. To allow comparison between recordings with different absolute signal levels, TMV and Ca^2⁺^ signals were aligned to their respective baseline values, setting all signals to a common reference point, while preserving the absolute magnitude of stimulus-induced changes. The resulting traces were subsequently low-pass filtered using a Butterworth filter to suppress high-frequency noise. Number of experiments: a: 6, b: 8. **c**–**e**: Representative examples showing synchronization between TMV and CaRh responses in S-HEK cells. Single AP for S-HEK cells was recorded after exposing S-HEK cells to one 450 V/cm 10 µs pulse (**c**), while multiple APs for S-HEK cells were recorded after exposing S-HEK cells to 400 V/cm 10 µs pulse (**d**). Prolonged depolarization without complex Ca^2⁺^ response was only observed in S-HEK cells at highest electric field, i.e., 600 V/cm (**e**). Number of experiments: c: 5, d: 15, e: 6. **f** Prolonged depolarizations without complex Ca^2⁺^ response were recorded after exposing NS-HEK cells to one 10 µs pulse at intermediate and high E, e.g. 450 V/cm. Number of experiments: **f**: 24. Arrows mark the pulse delivery. Note the different y axis ranges.

To elucidate whether the observed complex Ca^2⁺^ responses to single electric pulses are related to AP formation, we used an experimental setup that enabled simultaneous measurements of TMV and intracellular Ca^2⁺^. As in the experiments presented in Figs. 6 and 7, S-HEK and NS-HEK cells were exposed to single 10 µs monophasic pulses of increasing pulse amplitude (from 0 to 600 V/cm) with 2 min intervals. Consistent with the experiments in which Ca2⁺ alone was measured (Fig. 6), the complex Ca^2⁺^ response again appeared in S-HEK but not NS-HEK cells (Fig. 8a, b). In S-HEK cells, the APs elicited by a pulse coincided with corresponding peaks in intracellular Ca^2⁺^ (Fig. 8a), linking the multiphasic Ca^2⁺^ response to AP firing. Overall, the separate and simultaneous measurements of TMV and Ca^2⁺^ gave comparable results, with minor differences attributable to the different fluorescent indicators (ElectroFluor630 vs. FluoVolt for TMV and Fura-2 vs. Rhod-2 for Ca^2⁺^).

Whether the initial peak was followed by a higher, amplified peak a few seconds or a few tens of seconds later also depended on the amplitude of the delivered pulse. As in experiments presented in Figs. 6 and 7, the amplified peaks were observed at intermediate E. For example, at 400, 450 and 500 V/cm across 22 observations, we recorded single APs with amplified Ca^2⁺^ peak (Fig. 8c) in 5 instances (22.7%), while multiple APs with corresponding multiple amplified Ca^2⁺^ peaks (Fig. 8d) were detected 15 times (68.2%). Prolonged depolarizations, where the delivered electric pulse caused an initial spike in TMV and Ca^2⁺^ levels followed by a prolonged plateau phase in both TMV and Ca^2⁺^, occurred twice at 500 V/cm (9.1%). At higher electric fields (600 V/cm), the prolonged depolarizations and Ca^2⁺^ plateau occurred in all six experiments tested (Fig. 8e). In NS-HEK cells, we only detected prolonged depolarizations after pulse deliveries at intermediate and high E without the complex Ca^2+^ response observed in S-HEK cells (Fig. 8f).

## Discussion

### Spontaneous and stimulated multiple action potentials are linked to action potential propagation along S-HEK cell monolayer

In our previous studies^21–24^, we observed spontaneous APs in dense S-HEK monolayers, and multiple APs in response to a single electric pulse. We hypothesized that these two phenomena share the same underlying mechanism. To identify this mechanism, we considered two possibilities: (1) oscillatory behavior mediated by endogenous ion channels and electroporation-induced leak currents or (2) repeated activation of propagating AP waves within the electrically coupled cell monolayer.

The combination of K_ir_2.1 channels with leak conductance produces electrical bistability with two stable TMV states—a polarized resting state and a depolarized state—but not oscillatory behavior^19,20^. However, other endogenous voltage-gated ion channels could potentially enable single-cell oscillations. Spontaneous APs in neurons can arise from a steady depolarizing current and typically require the presence of K_V_ channels^46^. Similarly, multiple spontaneous APs observed after electroporation were attributed to the interplay between leak currents and L-type Ca_V_ channel activation^25,26^. Using ion channels inhibitors, we found that potassium channels targeted by TEA (excluding K_ir_ channels) were not involved in spontaneous AP generation. Ca_V_ channel inhibition by verapamil reduced or abolished spontaneous APs in some cultures but did not prevent pulse-triggered multiple APs. Note that verapamil also affects K_ir_ channels at higher concentrations, though our experiments used lower concentrations^39^.

In contrast, inhibition of gap junctional coupling with carbenoxolone (CBX) consistently terminated spontaneous APs in S-HEK monolayers and abolished multiple APs during the recording window (2.8 s). Moreover, CBX prevented AP propagation from the pulsed region to areas outside the electrodes, demonstrating that intercellular electrical coupling is required for the observed repetitive activity. At the molecular level, we confirmed that S-HEK cells endogenously express Cx43 and, to a lesser extent, Cx45, corroborating their electrical connectivity. Expression levels of these connexins did not differ statistically between spontaneously spiking samples and samples triggering multiple APs in response to pulses. These findings indicate that both spontaneous and pulse-triggered multiple APs primarily arise from AP propagation through the S-HEK monolayer rather than from single-cell oscillations.

This interpretation is consistent with previous optical mapping studies demonstrating that electrically coupled excitable HEK cells support propagating waves, including spiral waves^14–16^. Such wave dynamics can lead to repeated activation of a given region, which appears as periodic APs in our field of view. Drifts of spiral centers and chaotic patterns of colliding waves have also been observed^14,16^, which can explain the occasional non-periodic multiple APs we recorded. These spiral centers did not appear associated with morphological defects^16^; however, they could be anchored to small acellular obstacles^14^ or initiated by depolarization through light-activated ion channels^15^. Similarly, we observed that making a simple scratch provoked spontaneous APs. Moreover, applied electric pulses triggered multiple APs or sustained spontaneous APs that persisted for several minutes. Electroporation-induced membrane permeabilization may provide the depolarizing current that initiates propagating waves. We previously demonstrated that electroporation can be detected at electric field strengths close to the excitation threshold^21,22^.

To test whether S-HEK cells can generate multiple APs at the single-cell level, we performed additional experiments in which individual S-HEK cells were exposed to electric pulses (Suppl. Figs. S11, S12). We observed that the bistable nature of S-HEK cells allows spontaneous shifts between polarized and depolarized states even without pulse delivery (Suppl. Fig. S12). However, these shifts occur more slowly than the period of the multiple APs we observed (multiple seconds vs. a fraction of a second, see Suppl. Fig. S11). Applied electric pulses may further promote such shifts by providing depolarizing leak current through the electroporated membrane. While single-cell bistable shifts may contribute to AP initiation, the observed spontaneous and multiple APs are better explained by AP propagation, as they occur on a shorter time scale than bistable shifts.

What initiates the spontaneous depolarizations in the first place, however, remains to be determined. HEK293 cells express HCN2 and HCN3 at the mRNA level (Human Protein Atlas^41^, Table S2), which could in principle provide a pacemaker-like (“funny”) depolarizing current as in cardiac pacemaker cells. However, a consistent endogenous funny-like current has not been detected electrophysiologically in HEK293 cells^47^, so this possibility, which we did not test, remains uncertain.

### Multiphasic calcium response in S-HEK cells is linked to action potentials after pulse application

As we have shown previously^21,22^, S-HEK monolayers respond to applied electric pulses with a complex calcium response: after the initial peak in intracellular Ca^2+^, likely resulting mostly from electroporation, a second, much higher peak is observed (sometimes even additional peaks). In non-excitable NS-HEK cells, these biphasic or multiphasic Ca^2+^ responses were never observed: the cells responded only with the initial peak immediately after the pulse. In our present study, we aimed to elucidate the potential mechanism behind the Ca^2+^ response in S-HEK cells.

Our experiments with calcium chelation and store depletion revealed that all Ca^2+^ peaks originate from the extracellular medium. Whenever we pulsed cells in Ca^2+^-free medium (with the chelator EGTA), we did not record any Ca^2+^ response in either S-HEK or NS-HEK. Furthermore, depleting intracellular Ca^2+^ stores from the endoplasmic reticulum with thapsigargin, while maintaining Ca^2+^ in the extracellular medium, did not abolish the complex Ca^2+^ response in S-HEK cells. This demonstrates that the secondary peak does not result from calcium-induced Ca^2+^ release (CICR) that relies on internal Ca^2+^ stores^28^, but rather from continued influx across the plasma membrane.

From the experiments where TMV and internal Ca^2+^ were monitored simultaneously, it was clearly seen that additional APs cause corresponding peaks in the Ca^2+^ response. Thus, in cases where two or more Ca^2+^ peaks were observed, these peaks were a consequence of additional APs occurring after a single electric pulse exposure. This synchronization indicates that Ca^2+^ uptake is voltage-dependent and confirms our hypothesis that the complex Ca^2+^ response in S-HEK cells is related to the excitability of S-HEK cells.

The resting TMV differs between S-HEK and NS-HEK cells (approximately −70 mV and −20 mV, respectively)^15^, creating a higher electrochemical gradient for Ca^2+^ in S-HEK cells. This difference likely contributes to both the higher Ca^2+^ peaks in S-HEK cells compared to NS-HEK cells and the secondary peak phenomenon in S-HEK cells. After pulse application, the initial Ca^2+^ peak in both cell types can result from both electroporation and opening of voltage-gated calcium channels. In S-HEK cells, the AP causes membrane depolarization, which reduces the Ca2⁺ electrochemical gradient and slows Ca^2+^ uptake. When the membrane repolarizes after AP termination, the high electrochemical gradient is restored. Persistent electroporation then drives continued Ca^2+^ influx, producing the secondary peak. This mechanism is supported by the multiphasic Ca^2+^ responses accompanying multiple APs: each AP produces a corresponding Ca^2+^ peak, with uptake temporarily reduced during depolarization and restored upon repolarization (see Fig. 8). In NS-HEK cells, the higher (less negative) resting TMV creates a lower Ca^2+^ electrochemical gradient and slower dynamics in membrane potential changes (absence of APs). Consequently, we did not observe a complex Ca^2+^ response in NS-HEK cells under any conditions.

The voltage-dependent increase in intracellular Ca^2+^ also points toward involvement of voltage-gated calcium (Ca_V_) channels. Depolarization of the plasma membrane during APs can cause Ca_V_ opening with subsequent increase in intracellular Ca^2+^^48^. Ca^2+^ uptake through Ca_V_ channels in S-HEK cells may add up to Ca^2+^ uptake through the electroporated plasma membrane. This may additionally explain why Ca^2+^ peaks in S-HEK were consistently higher than in NS-HEK where APs did not occur (see Fig. 7). In NS-HEK cells, Ca_V_ channels are likely inactivated due to the less negative resting TMV, and thus depolarization caused by electroporation cannot activate them. In contrast, the more negative resting TMV in S-HEK cells allows Ca_V_ channels to remain available for activation during APs.

Our experiments with Ca_V_ blocker verapamil (Fig. 1) suggested that some of these channels also affect spontaneous/multiple APs. According to the Human Protein Atlas^41^ and Jin et al.^43^, the most highly expressed Ca_V_ channel in HEK293 cells is Ca_V_3.2; however, we detected relatively weak expression of these channels in our cells (Fig. 5). While low expression of endogenous ion channels in HEK cells is expected, we might have underestimated their expression for three reasons: (1) S-HEK cells derive from a different strain of HEK (HEK293T), which might preferentially express a different type of Ca_V_ channels, (2) expression of different proteins and lower resting TMV in S-HEK cells affects Ca_V_ expression, and (3) ineffective immunoblotting. Nevertheless, verapamil can affect other ion channels including K_ir_^39^, and its effect on APs might be related to this non-specificity.

When S-HEK monolayers were exposed to the gap junction inhibitor CBX, the complex Ca^2⁺^ response decreased but was not abolished (Fig. 7), and at the single-cell level the Ca^2⁺^ peaks persisted but became less synchronized than in control cells (Suppl. Fig. S9). The persistence of Ca^2+^ responses in cells exposed to CBX is consistent with other findings^45^. Because each Ca^2⁺^ peak coincides with an AP (Fig. 8), this suggests that gap junction coupling normally synchronizes the per-cell responses across the monolayer rather than generating them: uncoupling the cells desynchronizes the peaks—lowering the population-averaged response—without abolishing the underlying single-cell events. These persisting, desynchronized peaks may reflect individual cells spontaneously shifting between polarized and depolarized states (the bistable behavior described above), on a time scale consistent with the transitions shown in Suppl. Fig. S12.

Several other factors may also contribute to the residual response. CBX may not block gap junctions completely, so some slow AP propagation could persist even at conditions where we observed inhibition of APs propagation in TMV experiments. Our TMV experiments captured only 2.8 s time windows, which may have missed slower AP propagation events that could contribute to the complex Ca^2+^ response measured over 40 s. Gap-junction-mediated Ca^2+^ waves can amplify the response in monolayers, and CBX need not eliminate them, since such waves can also spread by paracrine signaling between adjacent cells^30^. CBX additionally blocks Ca_V_ channels in part^49^, which may contribute to the reduced Ca^2⁺^ response in both S-HEK and NS-HEK cells.

While our results link the complex Ca^2+^ response in S-HEK monolayers to propagating (multiple) APs, we cannot fully exclude a contribution from other Ca^2+^-handling pathways. Because Ca^2⁺^ acts as a signaling ion, its handling is intricate, involving numerous ion channels, pumps, and exchangers, as well as Ca^2+^-binding proteins and organelles that serve as buffers and internal stores^50–52^. A permeabilized plasma membrane provides an additional pathway for Ca^2+^ influx from extracellular medium. This Ca^2+^ influx may mimic a signal which can trigger a response through various calcium-based effectors, followed by different pathways of restoration of Ca^2+^ homeostasis^50–52^. Different Ca^2+^ responses to electric pulses in S-HEK and in NS-HEK cells may point to the differences in the Ca^2+^ handling pathways. Each cell type expresses a unique set of components from the calcium signaling systems with different spatial and temporal properties^50^. Some components may only be expressed /functioning in S-HEK but not in NS-HEK cells. Different protein expression and resting TMV in both types of cells can influence expression of other proteins^53,54^.

### Effects of electroporation on electrical coupling between cells

Our finding that multiple APs triggered by a single electric pulse are linked to AP propagation through gap junctions provides new insights into how electroporation affects electrical coupling between cells. Previous research demonstrated that sublethal exposure to 100 ns pulses at 10–20 kV/cm inhibits gap junctional intercellular communication, mediated by connexin Cx43, in rat liver epithelial cells within 15 min after pulse application^55^. Notably, these conditions produced no detectable propidium iodide uptake, suggesting that gap junction inhibition can occur at electric field strengths below those required for detectable cell membrane electroporation.

However, propidium iodide uptake is not a sufficiently sensitive indicator of electroporation. In our first study in S-HEK cells we found that AP prolongation caused by additional leak current through the electroporated membrane indicates electroporation at electric field strengths considerably lower than those required for propidium iodide uptake^21^. This demonstrates that electroporation-induced changes in cellular electrophysiology can occur at much milder conditions than detectable by propidium iodide.

In our previous studies, we observed multiple APs with prolonged durations characteristic of electroporation^21,22^. Since the present study demonstrates that multiple APs result from AP propagation through gap junctions, this suggests that plasma membrane electroporation occurs at lower electric field strength than gap junction inhibition, and APs altered by electroporation can propagate through gap junctions. If gap junction inhibition occurred at the same or lower electric field strengths as electroporation, prolonged APs would not be able to propagate through the monolayer, and we would not observe prolonged multiple APs. This is particularly evident for the shortest pulses (≤ 10 µs), where excitation was always accompanied by electroporation, yet multiple prolonged APs were still observed^22^.

Gap junction conductance depends on transjunctional voltage and intracellular calcium concentration^56^. Since electroporation causes substantial Ca^2+^ influx, as demonstrated in our calcium imaging experiments, sustained or more severe electroporation could eventually lead to gap junction block through calcium-mediated mechanisms. This may explain why gap junction inhibition was observed in rat liver epithelial cells exposed to high-amplitude nanosecond pulses^55^, where the electric field strengths (10–20 kV/cm) were considerably higher than those used in our experiments (max 2 kV/cm for 500 ns pulses)^22^.

These findings have implications for understanding acute electrophysiological effects during pulsed field ablation, particularly at lesion borders where cells may be reversibly electroporated^57^. Our results suggest that gap junctions in these border zones may remain functional despite electroporation, potentially allowing altered AP characteristics to propagate into adjacent tissue. In cardiomyocytes, an additional factor may also be at play: because they exhibit stronger calcium transients than S-HEK cells, electroporation-induced calcium overload may more readily contribute to gap junction dysfunction through calcium-dependent gating mechanisms. Intracellular calcium overload is a well-documented consequence of electroporation in cardiomyocytes^26^. Understanding the interplay between electroporation, gap junction function, and calcium handling will be important for interpreting the electrophysiological effects of pulsed field ablation in cardiac tissue.

## Conclusions

This study addressed two unresolved questions about S-HEK cell responses to electric pulses: the mechanism underlying multiple APs triggered by a single pulse, and the origin of complex calcium dynamics unique to S-HEK cells compared to their non-excitable NS-HEK counterparts. We demonstrated that multiple APs in S-HEK cells result primarily from intercellular propagation through gap junctions rather than single-cell oscillatory mechanisms. Furthermore, we showed that the complex, multiphasic calcium response in S-HEK cells reflects synchronized calcium influx during propagating APs, with extracellular calcium serving as the primary source rather than internal store release. Our study used CBX for gap junction inhibition. CBX is not fully selective for gap junctions, thus future studies using an independent assessment of intercellular coupling or another method of connexin disruption, such as genetic knockdown, would further strengthen our conclusions on the role of gap junctions.

Importantly, our findings suggest that electroporation in S-HEK cells occurs at lower electric field strengths than gap junction inhibition, enabling prolonged APs altered by electroporation to propagate through cell monolayers. This has implications for pulsed field ablation therapy, where reversibly electroporated cells at lesion borders may continue to transmit altered electrical signals through functional gap junctions. In cardiomyocytes, an additional factor may also be at play: because they exhibit stronger calcium transients than S-HEK cells, electroporation-induced calcium overload may more readily contribute to gap junction dysfunction, which needs to be explored in future studies. Overall, the results of our study advance our understanding of how electric pulses affect excitable tissues and may inform the development of more effective electroporation-based therapies for cardiac arrhythmias.

## Materials and methods

### Cells

An excitable, genetically engineered HEK cell line was developed and generously provided by the group of Adam E. Cohen at Harvard University^15,17^. These cells are now available from ATCC (cat. no. CRL-3479). The cell line was derived from HEK-293T cell line (ATCC CRL-3216). It was genetically modified to constitutively express Na_V_1.5 channels and with a tet-on doxycycline-induced expression of K_ir_2.1 channels. With doxycycline in the culture medium, we obtained excitable “spiking” S-HEK cells—cells with both Na_V_1.5 and K_ir_2.1 channels that can generate APs (“spikes”). Without doxycycline, cells grew as “non-spiking” NS-HEK cells with only Na_V_1.5 channels and served as complementary non-excitable counterparts.

The cells were propagated from week to week as NS-HEK cells, in Dulbecco′s Modified Eagle′s Medium high glucose growth medium (D5671, Sigma-Aldrich/Merck KGaA, Darmstadt, Germany), supplemented with 10% fetal bovine serum (F2442, Sigma-Aldrich/Merck), 2 mM glutamine (G7513, Sigma-Aldrich/Merck), penicillin–streptomycin (P07681, Sigma-Aldrich/Merck; penicillin 100 U/ml, streptomycin 100 µg/ml), 2 µg/ml puromycin (A1113803, Thermo Fisher Scientific Inc., Waltham, Massachusetts, U.S.), 5 µg/ml blasticidin (A1113903, Thermo Fisher Scientific), and 200 µg/ml geneticin (10131035, Thermo Fisher Scientific).

Two or three days prior to experiments, the cells were seeded to poly-L-lysine-coated 2-well Nunc Lab-Tek II Chambered #1.5 German Coverglass System (155379, Thermo Fisher Scientific) at a concentration 2.5 × 10^5^ or 10^5^ cells seeded per well, respectively. To obtain cell cultures with spontaneous APs the concentration was higher (5 × 10^5^ or 2.5 × 10^5^ cells per well, respectively), however, not all of these samples exhibited spontaneous APs when monitored for TMV changes^24^. For generation of S-HEK cells, 4 µg/ml doxycycline (D9891, Sigma-Aldrich/Merck) was added to cells when seeded. NS-HEK cells were seeded in culture medium without doxycycline. Passages lower than 15 were used in the experiments.

### Monitoring changes in transmembrane voltage

We monitored changes in TMV (including APs) optically, using a fluorescent potentiometric dye ElectroFluor630 (Di-4-ANEQ(F)PTEA, Potentiometric Probes, Farmington, CT, USA) and an epifluorescence microscope, as previously described^21,23^. Cells were labelled with 12 µM ElectroFluor630 dye in culture medium for 20 min in a refrigerator (4 °C). Afterwards, the cells were placed for 3 min in the incubator at 37 °C to facilitate warming to room temperature. The cells were then washed with Tyrode’s solution (2 mM KCl, 125 mM NaCl, 2 mM CaCl_2_, 1 mM MgCl_2_, 10 mM HEPES, 30 mM glucose, pH 7.3). Finally, a low K^+^ Tyrode’s solution (0.5 mM KCl, 126.5 mM NaCl, 2 mM CaCl_2_, 1 mM MgCl_2_, 10 mM HEPES, 30 mM glucose, pH 7.3) was added to cells. This solution was used in all TMV and Ca^2+^ experiments on both S-HEK and NS-HEK cells, based on our previous studies^21,22^. Its reduced K⁺ concentration supports robust AP triggering in S-HEK cells.

For monitoring APs and changes in TMV, cells were observed at room temperature under the microscope Thunder Imager Live Cell system for fluorescence microscopy. The microscope was equipped with DMi8 inverted fluorescent microscope, deep-cooled 4.2 MP sCMOS Leica DFC9000 Gt fast camera, a system of 8 LED diodes (LED8), and a UV light source (CoolLED pE340fura) (Leica Microsystems GmbH, Wetzlar, Germany). Cells were observed with a 40× objective and illuminated with excitation wavelength of 635 nm (50% LED power, exposure time of 10 ms, 4 × 4 binning). The emission was detected at around 700 nm wavelength using a DFT51010 filter set. Images in time-lapse mode were acquired using Leica Application Suite X (LAS X) version 3.10.1 software (Leica Microsystems, https://www.leica-microsystems.com/products/microscope-software/p/leica-las-x-ls/): one image every 36 ms, 80 images, 2.8 s total duration of image acquisition. If cells were exposed to electric pulses (in verapamil, CBX experiments), the pulse was delivered at 10th image (at around 324 ms, triggered by the TTL signal of the microscope triggering system). A time-lapse without pulse exposure recorded at the beginning of each experiment served as a control. When observing the cells exposed to electric pulses, we always chose a field of view in the middle between the electrodes, where the electric field is homogeneous.

We analyzed ElectroFluor630 fluorescence signal combined from the membranes of all the cells in the field of view, since with our method it is impossible to distinguish between two neighbouring cells and the signal from one cell is too weak to produce a valid result. Several hundreds of cells were present in the field of view, usually between 200 and 400. Image analysis was done in MATLAB version R2024b (Mathworks, Natick, MA, USA, https://ch.mathworks.com/products/matlab.html) to threshold the signal from the cell membranes, correct for photobleaching and extract relevant parameters (number of pulses, delay between pulse application and time of the first peak), as previously described^21–23^. The protocol is described in detail in our paper^23^, with visuals and a MATLAB algorithm attached. Briefly, images (see raw signal data curves in our previous reports)^22,23^ were thresholded to extract the time-dependent fluorescence signal from membranes in the entire captured image to improve the signal-to noise ratio. The extracted signals were also corrected for photobleaching and translated on y-axis such that the minimum value of each signal representing the relative fluorescence change (1–F/F0) was equal to 0. Signals were then processed using a custom MATLAB algorithm to determine the parameters of the signal: number of peaks, maximum response and delay between pulse application and time of the first peak. Graphs of all signals, annotated with the extracted parameters, were visually inspected to verify the correctness of the determined parameters.

### Monitoring intracellular calcium

Intracellular Ca^2+^ was monitored in ratiometric measurements with fluorescence microscopy using a fluorescent Ca^2+^ indicator Fura-2 acetoxymethyl (AM) ester (F1221, ThermoFisher Scientific)^21,58,59^. Cells were labelled with 2 µM Fura-2 AM in culture medium at 37 °C for 30 min, 3× washed with Tyrode’s solution and transferred to low K^+^ Tyrode’s solution.

Cells were observed at room temperature under Leica Thunder Imager Live Cell system with 63× objective. They were illuminated with UV light source (CoolLED pE340fura, Leica) at two excitation wavelengths (340 nm and 380 nm). For 340 nm excitation, exposure time was 200 ms, 2 × 2 binning, Fluorescence Intensity Manager (FIM) 100%, and for 380 nm excitation, exposure time was 25 ms, 2 × 2 binning, FIM 17%. The emission was detected at 510 nm wavelength using Leica Fura LED filter set. Images were acquired with the software LAS X (Leica) in time-lapse acquisition mode: one image every second, 40 images, 39 s total duration of image acquisition. The pulse was delivered at 5th image (at around 4 s, triggered by the TTL signal of the microscope triggering system). A time-lapse without pulse exposure recorded at the beginning of each experiment served as a control.

We analyzed Fura-2 fluorescence signal combined from the membranes of all the cells in the field of view. Several tens of cells were present in the field of view, usually between 70 and 90. For each time-lapse, the ratio images Fura-2 340/380 were calculated in Fiji (ImageJ) version 1.54p (National Institutes of Health, Bethesda, MD, https://fiji.sc) using the embedded function imageCalculator, as described before^22^. Each signal was baseline-corrected by subtracting its initial value, so that it started at 0 at t = 0 s. The location of peaks in each signal was identified using MATLAB’s built-in function findpeaks using a defined threshold prominence and a minimum distance between peaks and manually corrected, if necessary.

### *Simultaneous measurements of transmembrane voltage and internal Ca*^*2*+^

To elucidate whether the observed complex Ca2⁺ responses to single electric pulses are related to AP formation, we used an experimental setup that enabled simultaneous measurements of TMV and intracellular Ca^2^⁺. NS-HEK cells and S-HEK cells were prepared as described above. Experiments were performed two or three days after doxycycline treatment of S-HEK cells. On those days cells in Labtek chambers were first washed with Tyrode’s solution and then incubated in 600 µL of Tyrode’s solution per well containing fluorescent dyes for simultaneous measurements of TMV and internal Ca^2+^. Ca^2+^ indicator Rhod-2/AM (1 mM stock solution in DMSO) and voltage-sensitive indicator FluoVolt™ with Powerload™ (all from Thermo Fisher Scientific, USA), were diluted at 1:1000, 1:2000 and 1:100, respectively. Incubation lasted for 20 min at 37 °C in the dark. After the incubation period, cells were washed twice with Tyrode’s solution and afterwards fresh low K^+^ Tyrode’s solution was added to Labtek chambers.

Cells were imaged at room temperature using an inverted fluorescence microscope (Axiovert 200, Zeiss, Germany) with a × 63 objective and a system enabling simultaneous recording of APs and intracellular Ca^2^⁺ (IonOptix, USA). FluoVolt™ and Rhod-2/AM were excited at 470 nm and 565 nm, respectively, using a dual OptoLED power supply (Cairns Research, UK), and emissions were detected with photomultiplier tubes at 2 kHz. Data acquisition was performed using IonWizard version 7.8 software (IonOptix, USA, https://www.ionoptix.com/products/software/ionwizard-core-and-analysis/). Cells were exposed to single 10 µs electric pulses of increasing E every two minutes as described in our previous study^22^. E was estimated as the applied voltage-to-electrode-distance ratio of 240, 280, 300, 330, 360, 400, 450, 500 and 600 V/cm.

The results were expressed as ΔF/F₀. For TMV and Ca2⁺ in responses to 10 μs pulses of increasing electric field strength from representative experiments (Figs. 8a, b), TMV and Ca^2^⁺ signals were aligned to their respective baseline values, setting all signals to a common reference point, while preserving the absolute magnitude of stimulus-induced changes. The resulting traces were subsequently low-pass filtered using a Butterworth filter to suppress high-frequency noise.

### Pulse delivery

Single rectangle monophasic electric pulses of 10 µs, 100 µs and 1 ms were used in experiments (Suppl. Fig. S13, Table S1). Electric pulses were delivered by a prototype pulse generator^60,61^ connected to parallel Pt/Ir wire electrodes, with 0.8 mm diameter and 5 mm distance between inner edges, placed at the bottom of the Lab-Tek chamber. Voltage and current were monitored throughout the experiment with a WavePro 7300A oscilloscope, a differential voltage probe ADP305 and a current probe AP015, all from Teledyne LeCroy, Chestnut Ridge, NY, USA. The highest electric current flowing through the sample during experiments was 10 A (10 µs pulse, 2000 V/cm) and the energy of the pulse was 0.1 J. A “worst case” scenario of temperature rise in the medium between the electrodes without any heat dissipation^62^ was 0.136 K (10 µs pulse, 2000 V/cm).

### Experimental protocols

In this subsection we give further information on specific protocols for monitoring TMV and Ca^2+^ responses. In all experiments, cells were monitored under microscope at room temperature.

#### TEA and verapamil experiments (TMV response)

Tetraethylammonium (TEA, T2265, Merck, Darmstadt, Germany) is an inhibitor of potassium channels^63^ and verapamil (Verapamil hydrochloride, V4629, Merck) is an inhibitor of voltage-gated calcium channels^32^. S-HEK cell cultures with higher density for spontaneous APs in Labtek chambers were labelled with a fluorescent potentiometric dye ElectroFluor630 as described above and low K^+^ Tyrode’s solution was added to cells. The electrodes were fixed into the LabTek chambers and connected to the pulse generator. The cells were placed under the microscope for monitoring the changes of TMV. The changes of TMV before adding TEA or verapamil were monitored as a control and only samples with spontaneous APs were eligible for experiments in this section. When TEA was added to the cells by pipetting (the final concentration of 50 mM) the changes of TMV were monitored 5, 10 and 15 min after adding TEA. When verapamil was added to cells (the final concentration of 25 µM) the changes of TMV were monitored 5 and 10 min after adding verapamil. If spontaneous APs ceased a single electric pulse of 100 µs, 176 V/cm was applied during monitoring TMV to trigger APs.

#### Carbenoxolone experiments (TMV response)

Carbenoxolone (CBX, Carbenoxolone disodium salt, C4790, Merck) is a gap junction inhibitor^64^. S-HEK cell cultures with higher density for spontaneous APs in Labtek chambers were labelled with a fluorescent potentiometric dye ElectroFluor630 and low K^+^ Tyrode’s solution was added to cells. The cells were placed under the microscope, together with the electrodes connected to the pulse generator as in TEA experiments. The cells between the electrodes (i.e., the area with pulse exposure) were first monitored for changes in TMV before adding CBX by pipetting to obtain controls and determine if the cultures have spontaneous APs or not.

If the sample exhibited spontaneous APs, CBX was added to cells (the final concentration of 100 µM). The changes of TMV were recorded at 0 min, 2–3 min, 5 min, 7–8 min, 10 min, 15 min and 20 min after adding CBX. The same protocol was used also for control samples with adding only a vehicle (the same amount of ethanol as in CBX experiments, that is 0.4%).

If the sample did not exhibit spontaneous APs the cells without CBX were first exposed to a single electric pulse of 100 µs of increasing E every 2 min (126, 150, 176, 200 V/cm) until the pulse triggered multiple APs. The last pulse was later used in the rest of experiment. After that, 100 µM CBX was added to cells and the changes of TMV while exposed to a single electric pulse (100 µs, 126–200 V/cm) were recorded at 0 min, 2–3 min, 5 min, 7–8 min, 10 min, 15 min and 20 min after adding CBX. The same protocol was used also for control samples with adding only the vehicle.

If the electric pulses failed to trigger multiple APs, i.e., triggered only a single AP, the cells were moved in the position outside of the electrodes to examine AP propagation. Cells received 100 µM CBX and the changes of TMV while exposed to a single electric pulse (100 µs, a threshold amplitude for triggering APs while minimizing electroporation, i.e. 126–200 V/cm) were recorded before and at 0 min, 2–3 min, 5 min, 7–8 min, 10 min, 15 min and 20 min after adding CBX. The same protocol was used also for control samples with adding only the vehicle.

#### NS-HEK Calcium response and pulses with higher electric field experiments

NS-HEK cells were labelled with a fluorescent calcium indicator Fura-2, as described above, and low K^+^ Tyrode’s solution was added to the cells. The cells were placed under the microscope, together with the electrodes connected to the pulse generator. The cells were exposed to 10 µs and 1 ms electric pulses of higher E than in our previous study^22^. Each two min, a single pulse of increasing E was delivered to minimize the possible cumulative effects of consecutive pulses while keeping experiments short enough not to deteriorate the cells^21^. For 10 µs, E was estimated as the applied voltage-to-electrode-distance ratio of 600, 800, 1000, 1200, 1400, 1600, 1800 and 2000 V/cm, and for 1 ms, E was estimated as 180, 230, 280, 330, 380, 430, 480, 530, 580 and 800 V/cm. Intracellular Ca^2+^ was monitored as described above.

#### EGTA, Thapsigargin experiments (calcium response)

S-HEK and NS-HEK cells were labelled with a fluorescent calcium indicator Fura-2, as described above. The cells were placed under the microscope, together with the electrodes connected to the pulse generator. For EGTA experiments, the cells were exposed to electric pulses in low K^+^ Tyrode’s solution without Ca^2+^ supplemented with 5 µM EGTA. For thapsigargin experiments, the cells were labeled with Fura-2 in the presence of 200 nM thapsigargin (T7459, Thermo Fisher Scientific) for 30 min at 37 °C to empty internal stores of Ca^2+^. After the incubation, cells were washed and exposed to electric pulses in low K^+^ Tyrode’s solution. For combined experiments (both EGTA and thapsigargin), the cells were labeled with Fura-2 in the presence of 200 nM thapsigargin for 30 min, washed and exposed to electric pulses in low K^+^ Tyrode’s solution without Ca^2+^ supplemented with 5 µM EGTA. The cells were exposed to single 10 µs electric pulses of increasing E every two min (18 min in total) as described in our previous study^22^. E was estimated as the applied voltage-to-electrode-distance ratio of 240, 280, 300, 330, 360, 400, 450, 500 and 600 V/cm. Intracellular Ca^2+^ was monitored as described above. The same protocols were used also for control samples without pulse exposure.

#### Carbenoxolone experiments (calcium response)

S-HEK and NS-HEK cells were labeled with a fluorescent calcium indicator Fura-2, as described above. 100 µM carbenoxolone (CBX) was added to low K^+^ Tyrode’s solution and incubated for 15 min at room temperature to inhibit gap junctions. During this time, the cells were placed under the microscope, together with the electrodes connected to the pulse generator. Cells were exposed to single 10 µs electric pulses of increasing E every two min (18 min in total) as described in our previous study^22^. E was estimated as the applied voltage-to-electrode-distance ratio of 240, 280, 300, 330, 360, 400, 450, 500 and 600 V/cm. The last pulse (600 V/cm) was delivered 31 min after CBX was added to cells. Intracellular Ca^2+^ was monitored as described above. Control experiments without electric pulses were performed the same way as with pulses (image acquisition every two minutes). In control experiments without CBX, only vehicle was used (0.4% ethanol).

### Immunofluorescence

The NS-HEK cells were seeded onto poly-L-lysine-coated 15 mm #1.5 coverslips (1NT4.1, Carl Roth, Karlsruhe, Germany) at a concentration 3 × 10^4^ cells per coverslip. For generation of S-HEK cells, 4 µg/ml doxycycline (D9891, Sigma-Aldrich/Merck) was added to cells when seeded. NS-HEK cells were seeded in culture medium without doxycycline. Two days later, cells were briefly washed by DPBS (9130.2, Carl Roth, Karlsruhe, Germany), fixed with 4% paraformaldehyde in DPBS for 15 min, permeabilised by 0.1% Triton-X100 (28,314, Thermo Fisher Scientific) in DPBS for 10 min and washed with DPBS three times. Coverslips were then incubated overnight at 4 °C with the appropriate primary antibodies (listed in Table 1) diluted 1:100, while Cx43 antibody was diluted 1:500. The next day, coverslips were rinsed and incubated with corresponding secondary antibodies conjugated with Alexa Fluor 488, 546 or 647 (1:500, Thermo Fisher Scientific) for 2 h at room temperature. The nuclei were labelled by DAPI (1 µg/mL, Molecular Probes, Eugene, OR, USA) for 15 min. Following washing, the coverslips were mounted in ProLong Diamond (Molecular Probes, Eugene, OR, USA) and observed under the fluorescence microscope Elyra 7 (Zeiss, Oberkochen, Germany) by using a Plan-Apochromat 40x/1.4 oil DIC M27 objective and ZEN Black version 3.0 SR software (https://www.micro-shop.zeiss.com/en/de/softwarefinder/software-categories/zen-black). Three different biological replicates were stained and at least 5 different fields of view were imaged for each primary antibody. For a given staining, the images were acquired with the same settings.

**Table 1:** List of primary antibodies used in this study.

Antibody	Host	Type	Dilution	Supplier	Cat. No
Nav1.5	Rabbit	Monoclonal	1:2000	CST	#14421S
Cav3.2	Rabbit	Polyclonal	1:500	TFS	#PA5-106,771
Kir2.1(new)	Rabbit	Polyclonal	1:2000	TFS	#PA5-37,452
Kir2.1 (old)	Mouse	Monoclonal	1:2000	TFS	#MA5-27,681
Connexin (Cx45)	Mouse	Monoclonal	1:500	TFS	#41–5800
Connexin (Cx43)	Rabbit	Monoclonal	1:500	CST	#83,649

CST: Cell Signaling Technology; TPS: Thermo Fisher Scientific.

Adult cardiomyocytes were isolated from adult rats as described previously^65,66^. All animal procedures conformed to institutional and EU guidelines (Directive 2010/63/EU) and were approved by the Ethics Committee for Animal Experiments at UVHVVR (Slovenian Administration for Food Safety, Veterinary Sector and Plant Protection), license no. U34401-13/2021/8. Cells were fixed one hour after plating on PLL coated coverslips and labelled as described above for HEK cells.

### Immunoblotting

NS-HEK and S-HEK cells were cultured as described above. On the day of experiments cells were tested for spontaneous AP generation, as explained above. Afterwards, Tyrode’s buffer was removed in cells were rinsed three times with ice-cold 1× Phosphate Buffered Saline (PBS) buffer – 137 mM NaCl (SI-71382, Merck), 2.7 KCl (1049360550, Merck), 10 mM Na_2_HPO_4_ (1065860500, Merck) and 1.8 KH_2_PO_4._ (1048730250, Merck). The samples were then stored at –80 °C for later immunoblot analysis.

For protein extraction, cells were lysed using Pierce™ RIPA buffer (89901, Thermo Fisher Scientific), and total protein concentration was determined using the Pierce BCA Protein Assay Kit (23225 and 23227, Thermo Fisher Scientific) according to the manufacturer’s instructions. All samples were adjusted to the same protein concentration prior to gel electrophoresis.

Proteins were separated on Bolt™ 4–12% Bis–Tris Mini Protein Gels (1.0 mm; NW04125BOX, Thermo Fisher Scientific) using a Mini Gel Tank (A25977, Thermo Fisher Scientific) and transferred onto PVDF membranes with Power Blotter Select Transfer Stacks (Mini; PB5210, Thermo Fisher Scientific) using the Power Blotter Semi-Dry Transfer System, following the manufacturer’s protocols. To verify transfer efficiency and uniform loading, membranes were stained with Ponceau C Staining Solution (A40000278, Thermo Fisher Scientific) and scanned. Membranes were cut horizontally after Ponceau S staining and prior to hybridization with antibodies to allow for the detection of several different proteins on one membrane.

Membranes were then blocked for 1 h at room temperature in Tris-buffered saline with 0.02% Tween-20 (TBST; 20 mM Tris, 150 mM NaCl, pH 7.5) containing 3% bovine serum albumin (BSA; A2153, Merck). After blocking, membranes were incubated overnight at 4 °C with the appropriate primary antibodies (listed in Table X) diluted in TBST containing 3% BSA.

After washing with TBST, membranes were incubated with horseradish peroxidase (HRP)-conjugated secondary antibodies diluted in TBST with 3% BSA for 1 h at room temperature. Following additional washes, protein bands were detected using the Pierce™ Enhanced Chemiluminescence (ECL) reagent (32106, Thermo Fisher Scientific) and imaged with the Uvitec Alliance Q9 system (Uvitec, Cambridge, UK). Densitometric analysis was performed using Quantity One 1-D Analysis Software version 4.6.8 (Bio-Rad, Hercules, CA, USA, https://www.bio-rad.com/en-si/product/quantity-one-1-d-analysis-software?ID=1de9eb3a-1eb5-4edb-82d2-68b91bf360fb).

### Statistical analysis

Statistical analysis was performed by using Microsoft Excel for Microsoft 365, version 2606 (Microsoft, Redmond, WA, USA, https://www.office.com) and SigmaPlot version 15 (Systat Software, Chicago, IL, USA, https://grafiti.com/sigmaplot-detail/). Shapiro–Wilk normality test, Brown-Forsythe equal variance test, Kruskal–Wallis One Way Analysis of Variance on Ranks followed by Dunn’s or Tukey’s test for multiple comparisons, One-way ANOVA with Bonferroni’s test, Fischer’s exact test, Mann–Whitney Rank Sum Test for statistical differences.

## Supplementary Information

Supplementary Information 1

Supplementary Information 2

## Data Availability

The data presented in this study is available on request from the corresponding author.
