# Supplementary material for: Multiple action potentials and complex calcium dynamics following electroporation of S-HEK cells depend on gap junction coupling
Source: Sci Rep. 2026 Jul 28;16:34459. doi: 10.1038/s41598-026-63712-8 (PMC13710161; doi:10.1038/s41598-026-63712-8)
Supplement: Supplementary file 1 [file 41598_2026_63712_MOESM1_ESM.pdf]

RAW DATA OF IMMUNOBLOTTING Figs. 5 and S4

Multiple action potentials and complex calcium dynamics following electroporation of S-HEK cells depend on gap junction coupling

Tina Batista Napotnik[1], Anja Blažič[1], Vid Jan[1], Klara Bulc Rozman[1], Rok Šmerc[1], Lea Rems[1]*

[1] University of Ljubljana, Faculty of Electrical Engineering, Tržaška cesta 25, 1000 Ljubljana, Slovenia

Figure 5

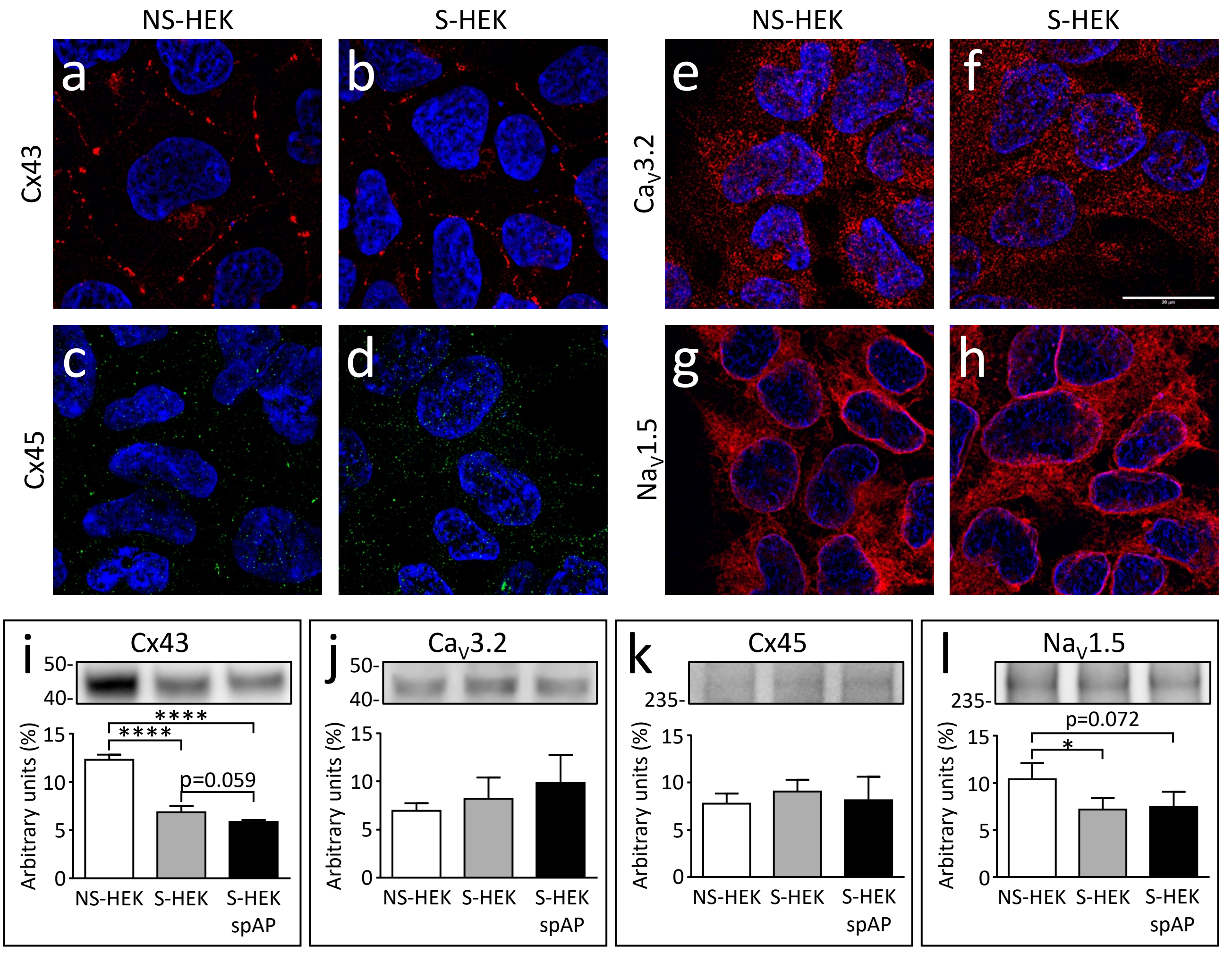

NS-HEK
S-HEK
NS-HEK
S-HEK
a
b
e
f
Cx43
$Ca_V3.2$
c
d
g
h
Cx45
$Na_V1.5$
i
Cx43
50-
40-
****
****
p=0.059
Arbitrary units (%)
15
10
5
0
NS-HEK
S-HEK
S-HEK spAP
j
$Ca_V3.2$
50-
40-
Arbitrary units (%)
15
10
5
0
NS-HEK
S-HEK
S-HEK spAP
k
Cx45
235-
Arbitrary units (%)
15
10
5
0
NS-HEK
S-HEK
S-HEK spAP
l
$Na_V1.5$
235-
p=0.072
*
Arbitrary units (%)
15
10
5
0
NS-HEK
S-HEK
S-HEK spAP

Figure S4

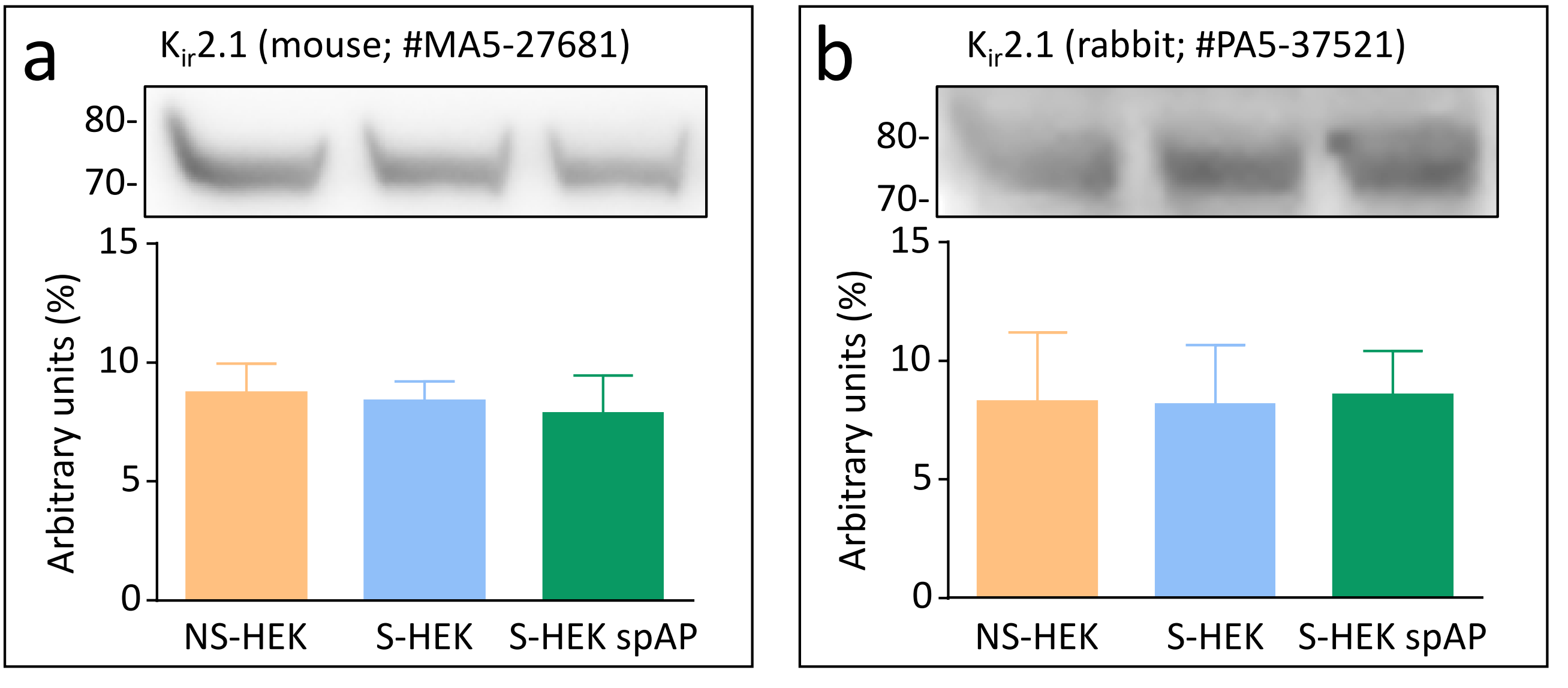
a
K_{ir}2.1 (mouse; #MA5-27681)
80-
70-
15
10
5
0
Arbitrary units (%)
NS-HEK
S-HEK
S-HEK spAP
b
K_{ir}2.1 (rabbit; #PA5-37521)
80-
70-
15
10
5
0
Arbitrary units (%)
NS-HEK
S-HEK
S-HEK spAP

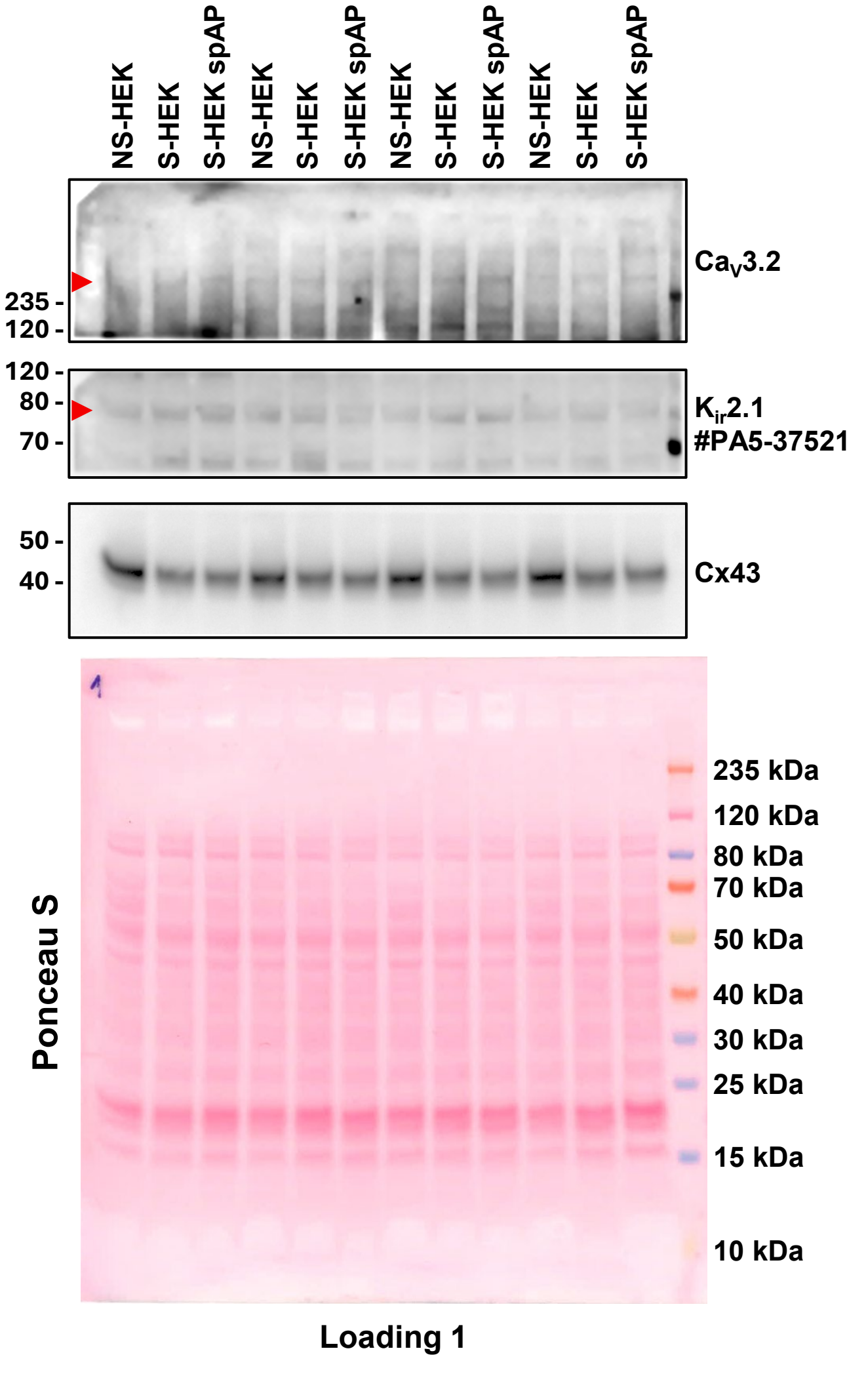

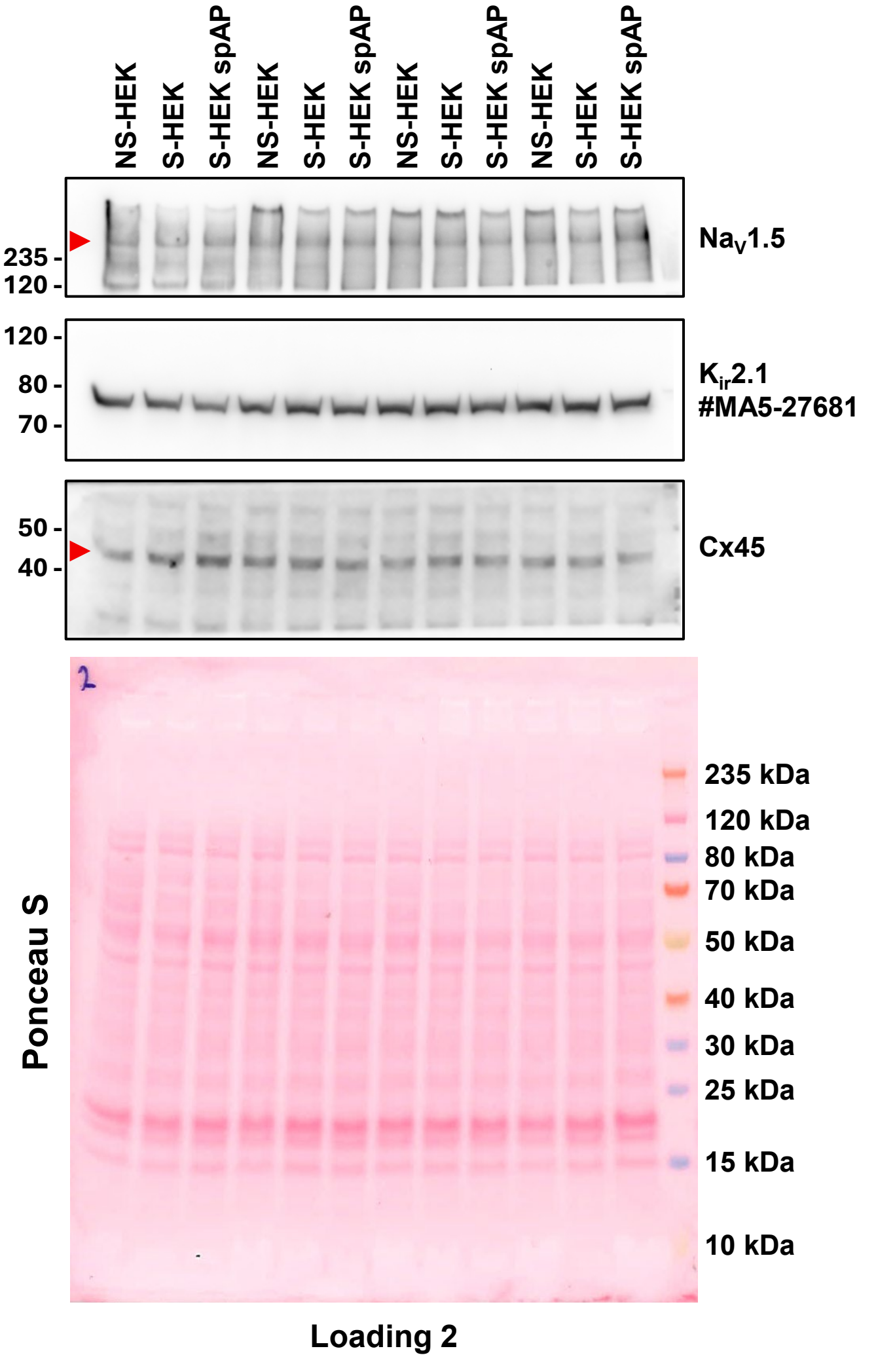

In case of unspecific bands red arrowhead marks the specific bands that were used in densitometric analyses
Membranes were cut horizontally after Ponceau S staining and prior to hybridization with antibodies to allow for the detection of several different proteins on one membrane.
