# Supplementary material for: Multiple action potentials and complex calcium dynamics following electroporation of S-HEK cells depend on gap junction coupling
Source: Sci Rep. 2026 Jul 28;16:34459. doi: 10.1038/s41598-026-63712-8 (PMC13710161; doi:10.1038/s41598-026-63712-8)
Supplement: Supplementary file 2 [file 41598_2026_63712_MOESM2_ESM.pdf]

Supplementary information 1

Multiple action potentials and complex calcium dynamics following electroporation of S-HEK cells depend on gap junction coupling

Tina Batista Napotnik, Anja Blažič, Vid Jan, Klara Bulc Rozman, Rok Šmerc, Lea Rems

University of Ljubljana, Faculty of Electrical Engineering, Tržaška cesta 25, 1000 Ljubljana, Slovenia

Supplementary figures

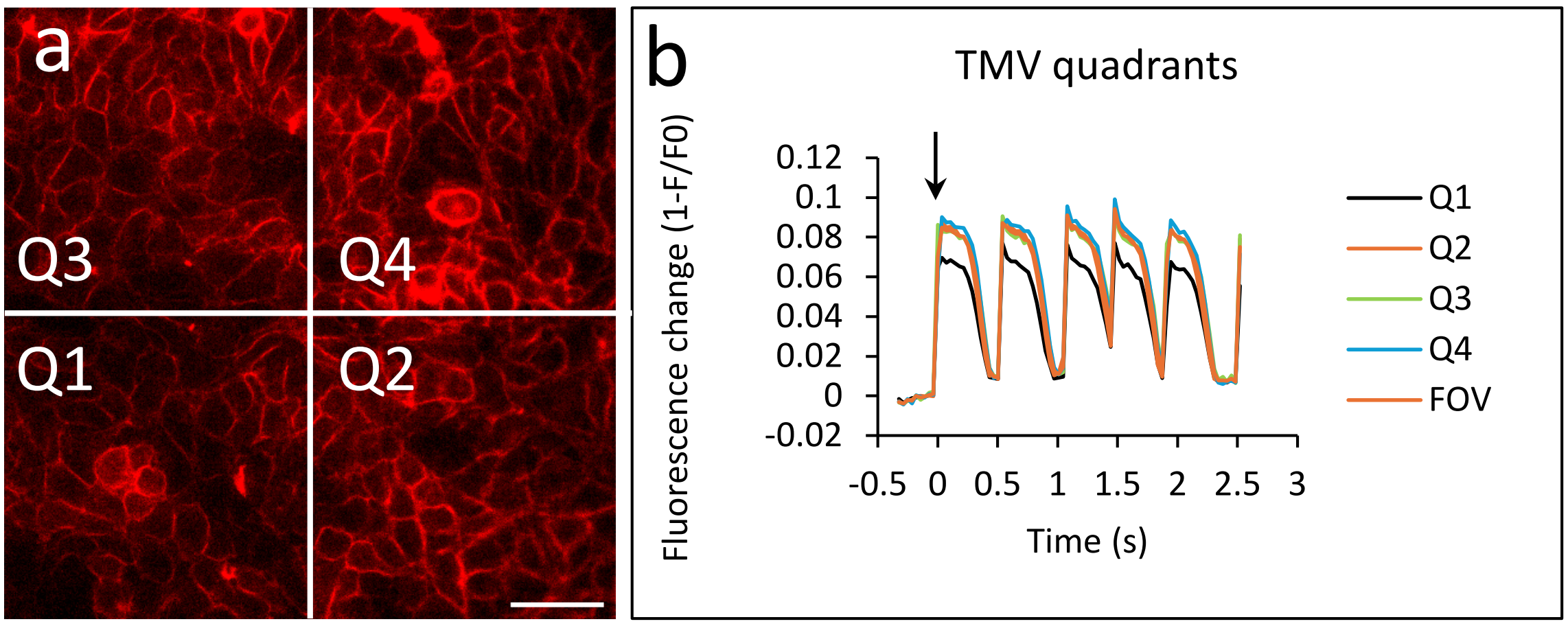

Figure S1: TMV signals in four separate quadrants of the whole field of view (FOV) matched the collective signal from the whole FOV. Cells were labelled with a potentiometric dye ElectroFluor630 and observed under a fluorescence microscope (**a**). Four of the quadrants (Q1-4) in a representative experiment that were used for further analysis are shown. Scalebar: 50 μm. (**b**). Cells were exposed to a single electric pulse of 100 μs, 176 V/cm, at time 0 s (arrow), that provoked multiple action potentials (APs) in cells. We have analyzed other 35 experiments (control, verapamil, CBX) and, in all cases, without exception, the signals from separate quadrants matched the signal from the whole FOV.

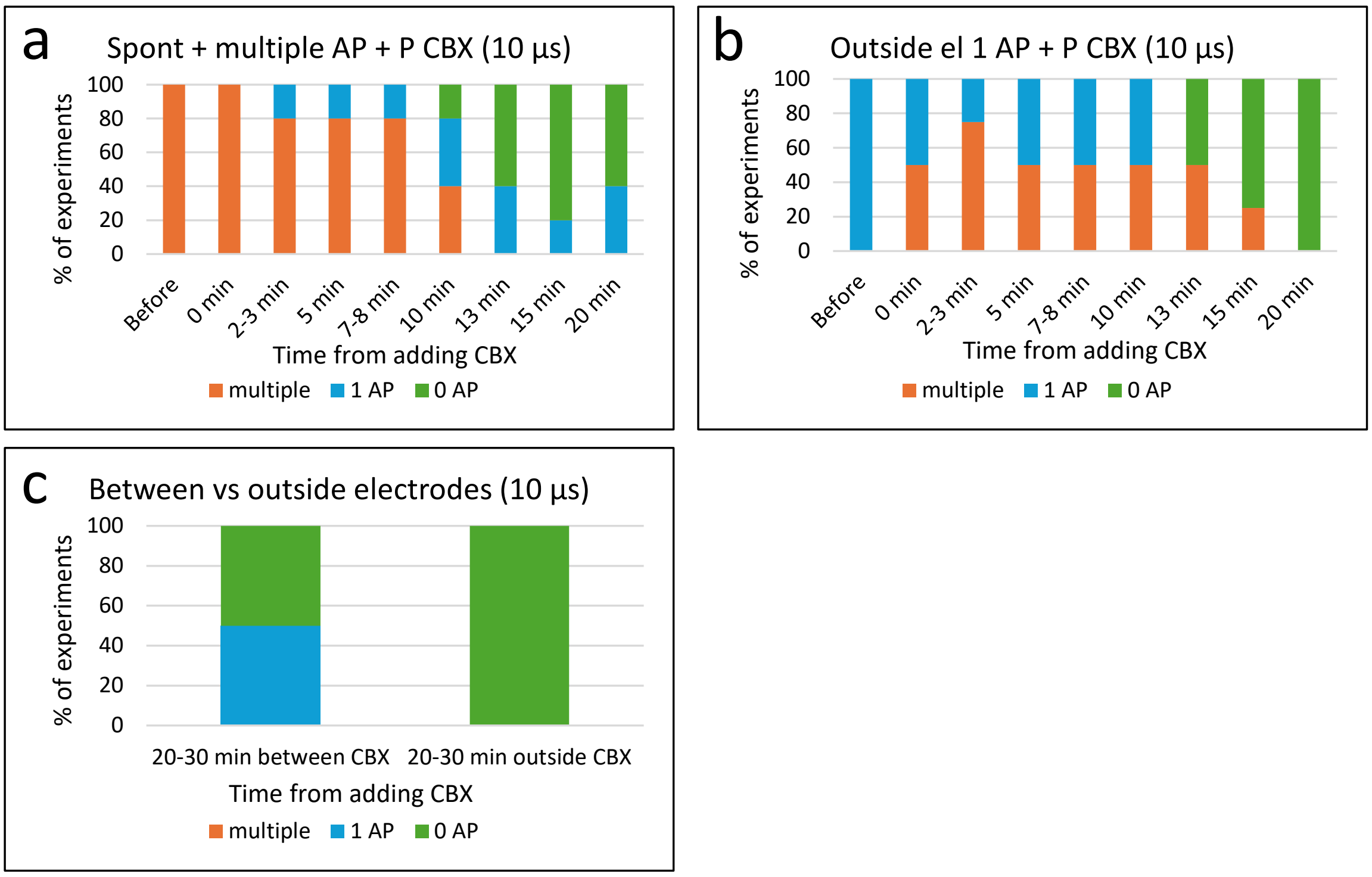

Figure S2: The effect of carbenoxolone (CBX) on triggering multiple AP and propagation of AP in S-HEK cell cultures, using 10 µs, 360-400 V/cm electric pulses. Different TMV responses were observed between the electrodes in cell cultures with spontaneous AP or triggered with a single electric pulse (**a**), or outside the electrodes in cell cultures, exhibiting only one AP (**b**). A comparison of different TMV responses between the electrode or outside 20 min after adding CBX is presented in (**c**). Number of experiments (N): **a**: 5, **b**: 4, **c**: 4.

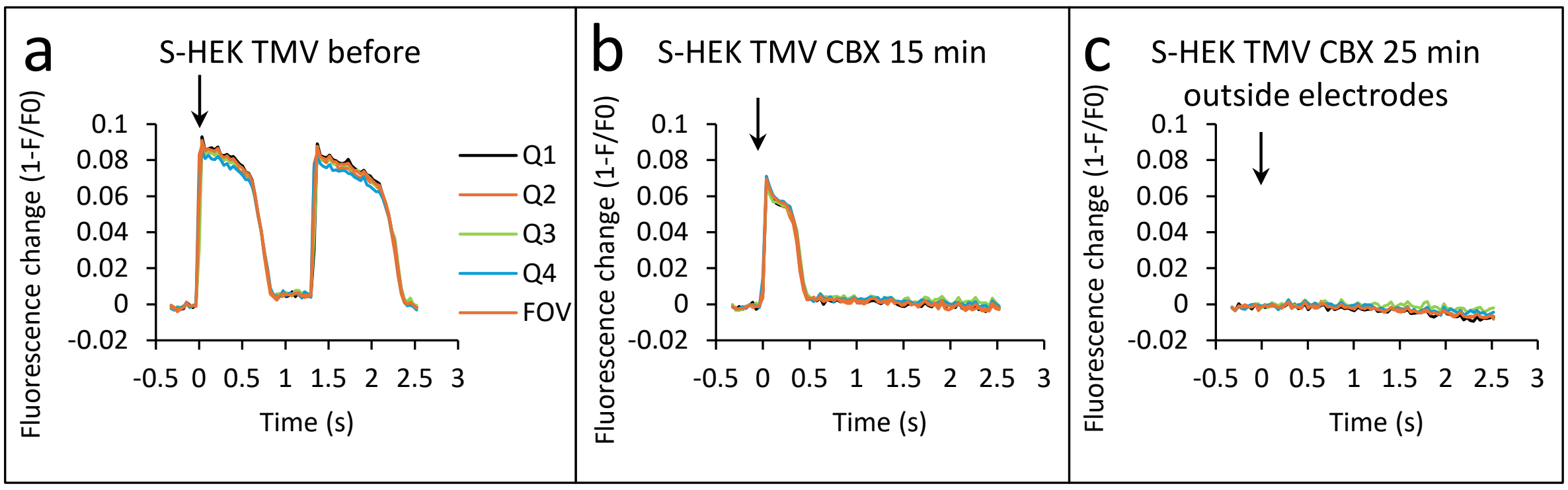

Figure S3: TMV signals from four quadrants of the field of view (FOV) in a representative experiment where S-HEK cells were treated with carbenoxolone (CBX). **a**: TMV signal in cells before the experiment. Cells were exposed to a single electric pulse of 100 μs, 176 V/cm, at time 0 s (arrows), that provoked multiple action potentials (APs) in cells. **b**: The same cells were incubated for 15 min in CBX and exposed to the same electric pulse as in **a**. Only one AP appeared. **c**: The cells outside the electrodes in the same experiment did not respond to electric pulse (same parameters as in **a**, **b**) after 25 min of CBX incubation. The traces from separate quadrants (Q1-4) matched the signal from the whole FOV.

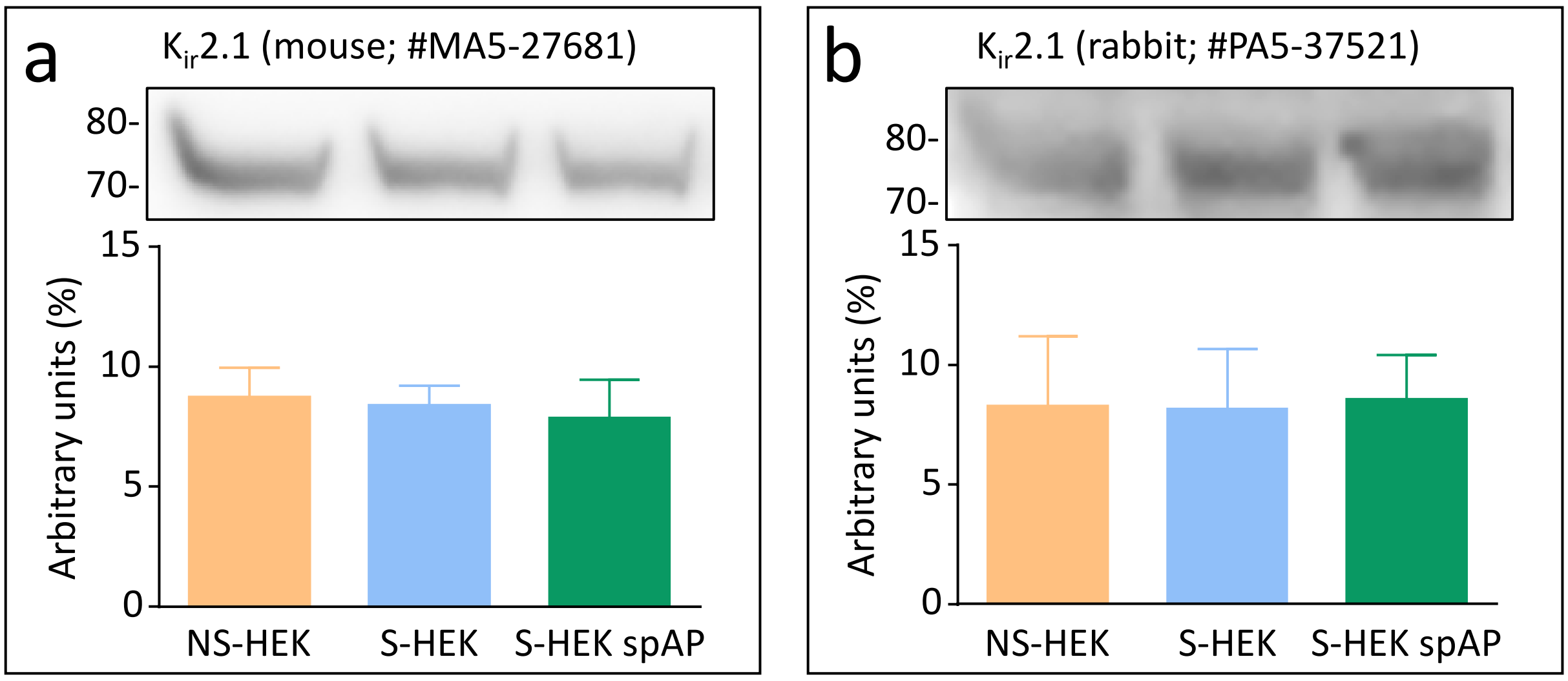

Figure S4: Protein expression of inward rectifying potassium channels (Kir2.1) in excitable and non-excitable HEK cells. **a**, **b**: Two different antibodies were used for immunoblotting to estimate protein abundance of $K_{ir}2.1$ in non-excitable (NS-HEK), excitable without spontaneous action potentials (S-HEK), and excitable HEK cells with spontaneous action potentials (S-HEK spAP). Monoclonal mouse (#MA5-27681; **a**) and polyclonal rabbit (#PA5-37452; **b**) primary antibodies were used. Number of experiments (N): 4.

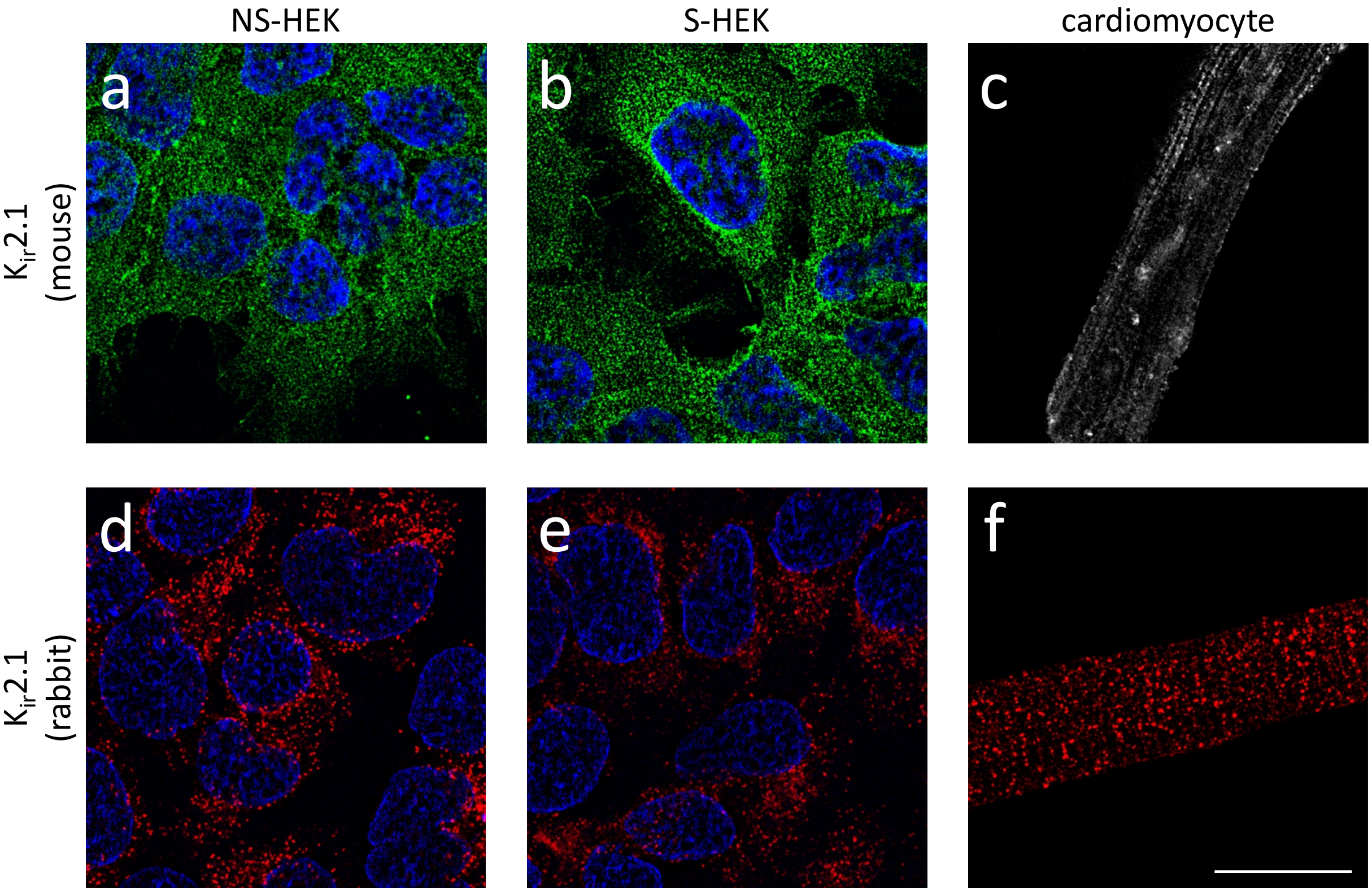

Figure S5: Protein expression of inward rectifying potassium channels ($K_{ir}2.1$) in non-excitable and excitable HEK cells. Two different antibodies raised against $K_{ir}2.1$ were used for immunofluorescent labelling of $K_{ir}2.1$ in (**a**, **d**) non-excitable (NS-HEK) and (**b**, **e**) excitable S-HEK cells, while (**c**, **f**) adult rat ventricular cardiomyocytes served as a positive control. Although we detected fluorescent signal in both HEK cell lines, the lack of ordered signal in cardiomyocytes indicates that the labelling is not specific to $K_{ir}2.1$. Number of experiments (N): 3. Scale bar, 20 µm.

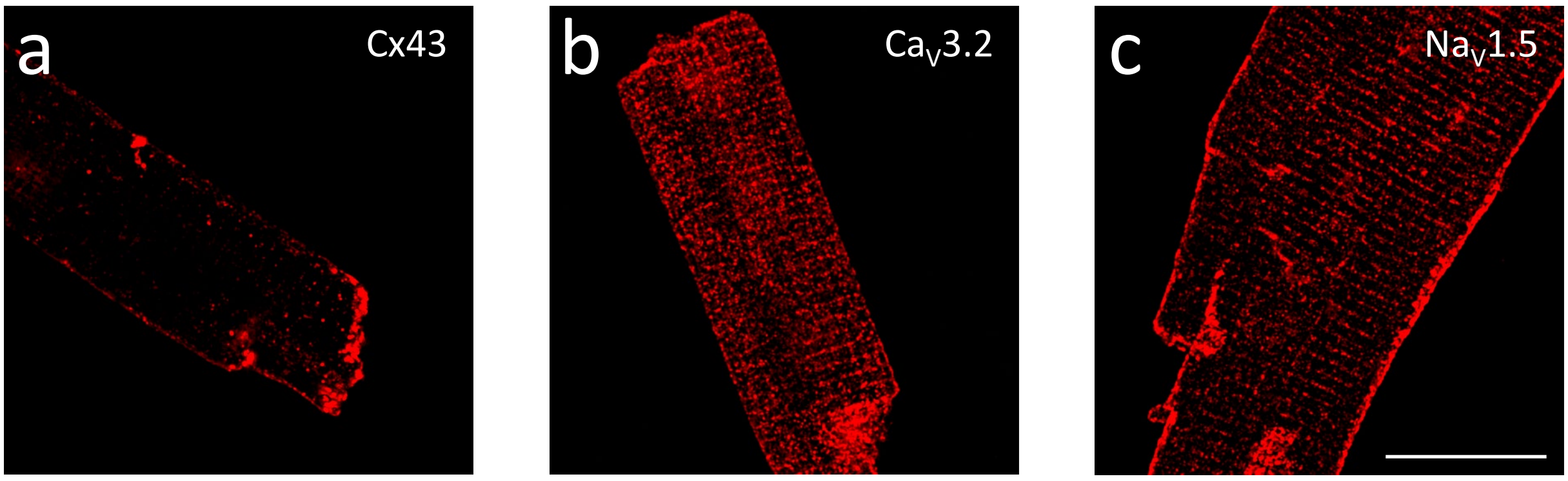

Figure S6: Antibodies for (**a**) Cx43, (**b**) $Ca_V3.2$ and (**c**) $Na_V1.5$ were validated on adult rat cardiomyocytes. Cx43 labeling is intense at cell junctions, as expected. $Ca_V3.2$ and $Na_V1.5$ are localized at lateral membranes, t-tubules and intercalated discs of cardiomyocytes. Therefore, the staining pattern corresponds to expected locations.

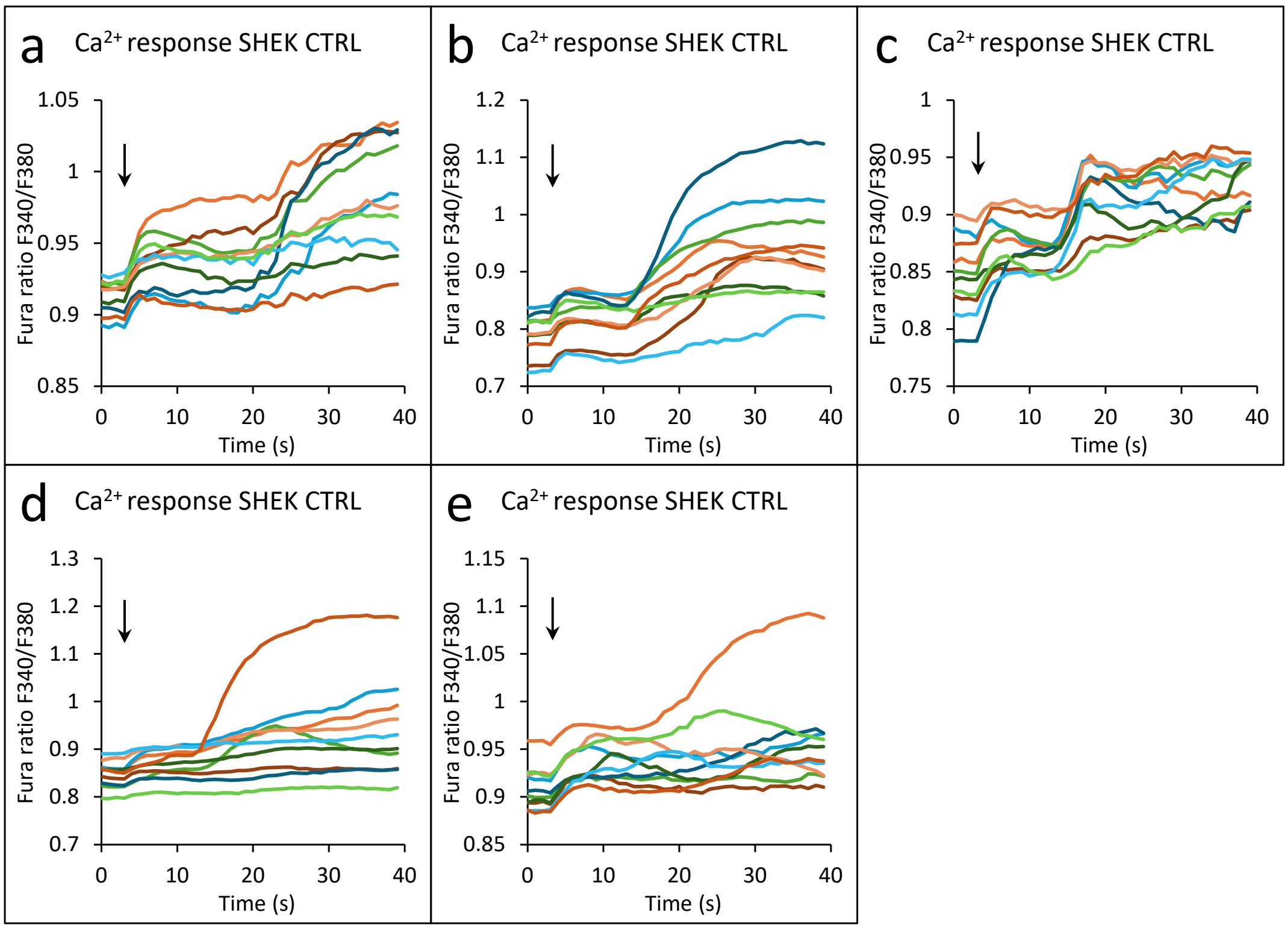

Figure S7: Fura-2 signal (Ca^{2+} response) in ten individual S-HEK cells at one pulse amplitude. (**a**-**e**) Each panel corresponds to a different experiment. A pulse was delivered at around 4th second after the image acquisition started (arrows). We analyzed pulse amplitudes that triggered a complex Ca^{2+} response in cell monolayers (400-500V/cm). All the cells have two or multiple Ca^{2+} peaks. Note the different y axis ranges.

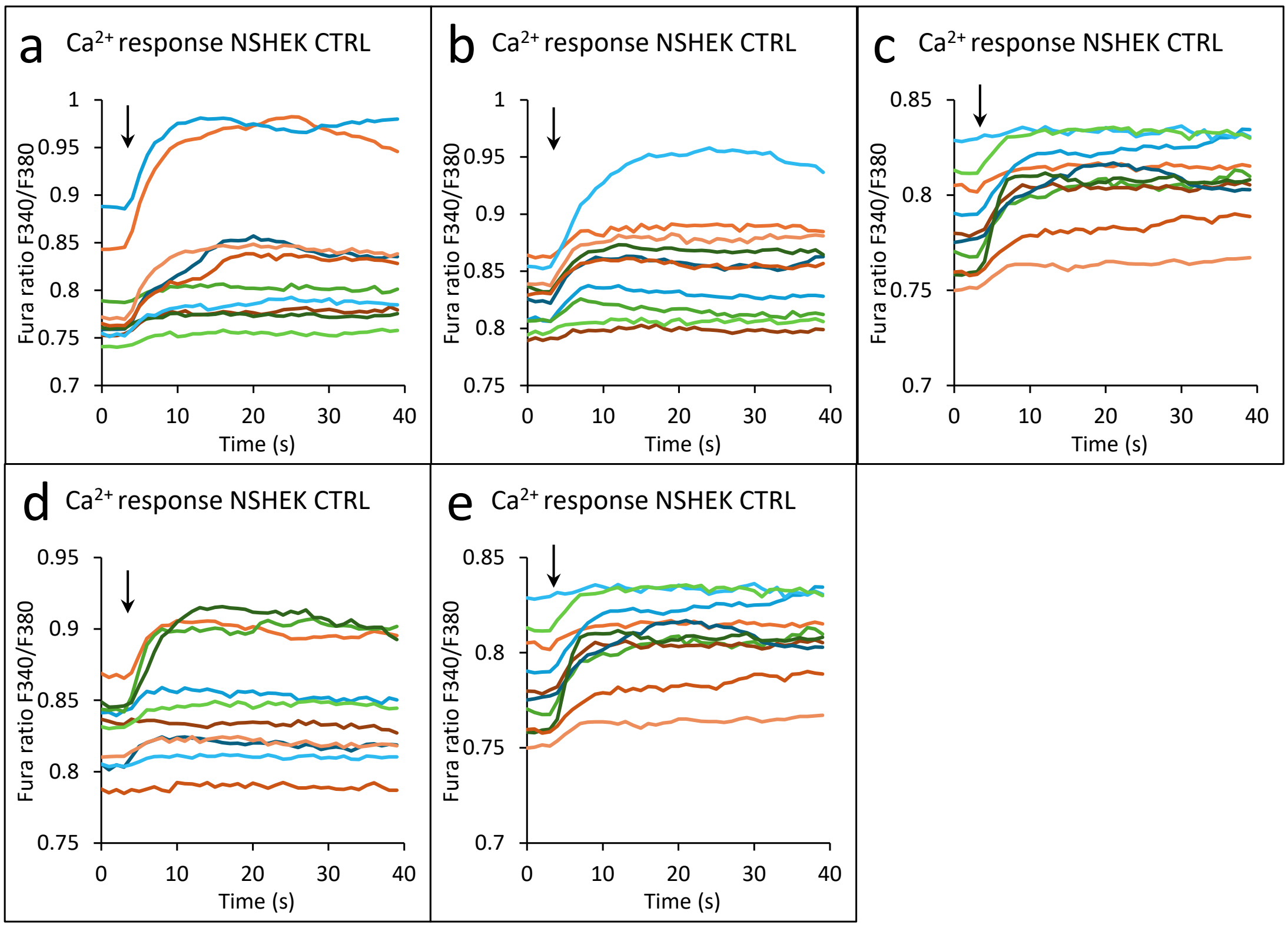

Figure S8: Fura-2 signal (Ca^{2+} response) in ten individual NS-HEK cells at one pulse amplitude. (**a-e**) Each panel corresponds to a different experiment. A pulse was delivered at around 4th second after the image acquisition started (arrows). We analyzed similar amplitudes as in Fig. S7 (400-500V/cm). All the cells have one Ca^{2+} peak. Note the different y axis ranges.

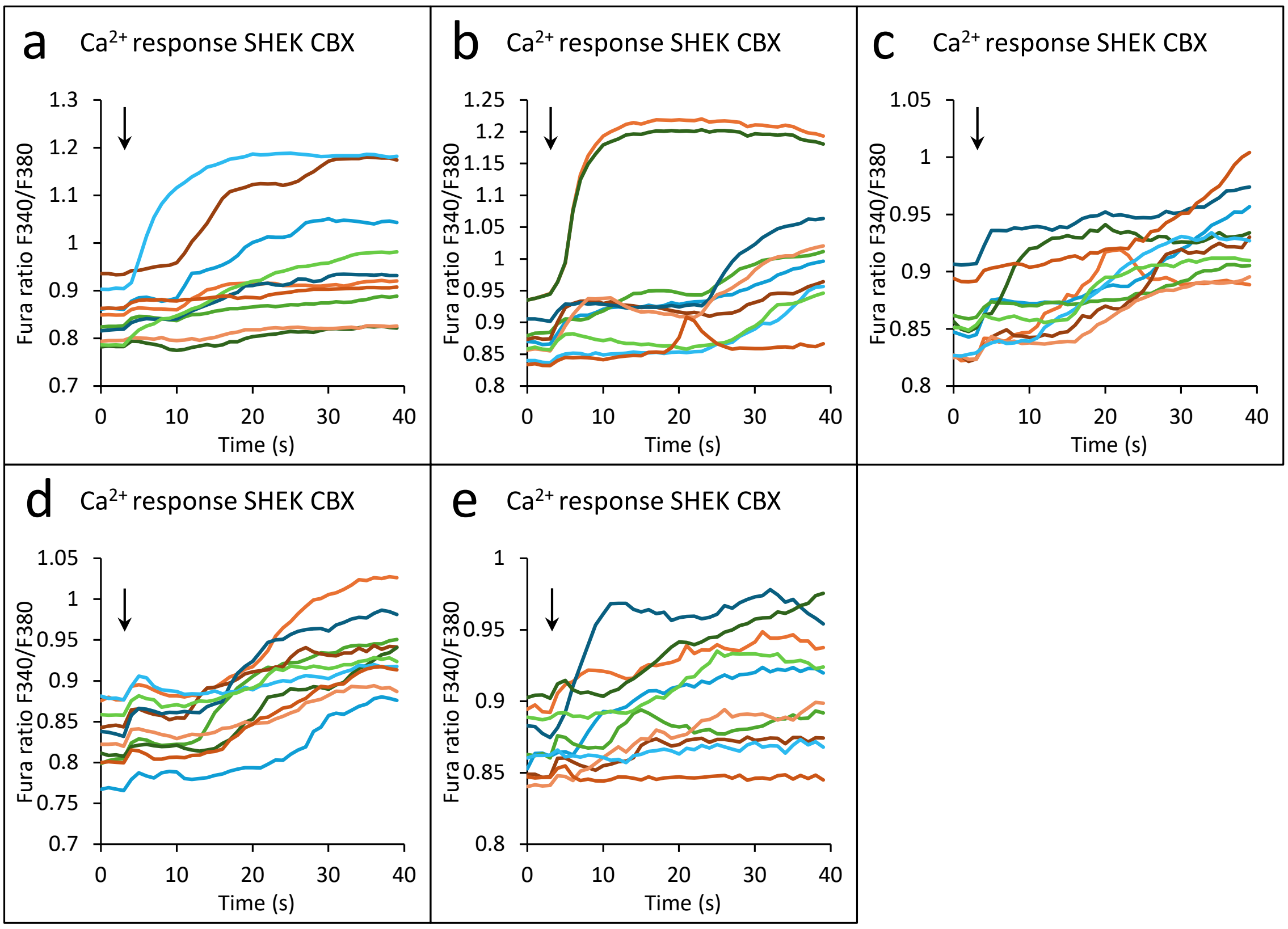

Figure S9: Fura-2 signal (Ca^{2+} response) in ten individual CBX-treated S-HEK cells at one pulse amplitude of five experiments (**a-e**). A pulse was delivered at around 4th second after the image acquisition started (arrows). We analyzed the amplitudes that triggered a complex Ca^{2+} response in cell monolayers (450-600V/cm). Most of the cells have two or multiple Ca^{2+} peaks. Note the different y axis ranges.

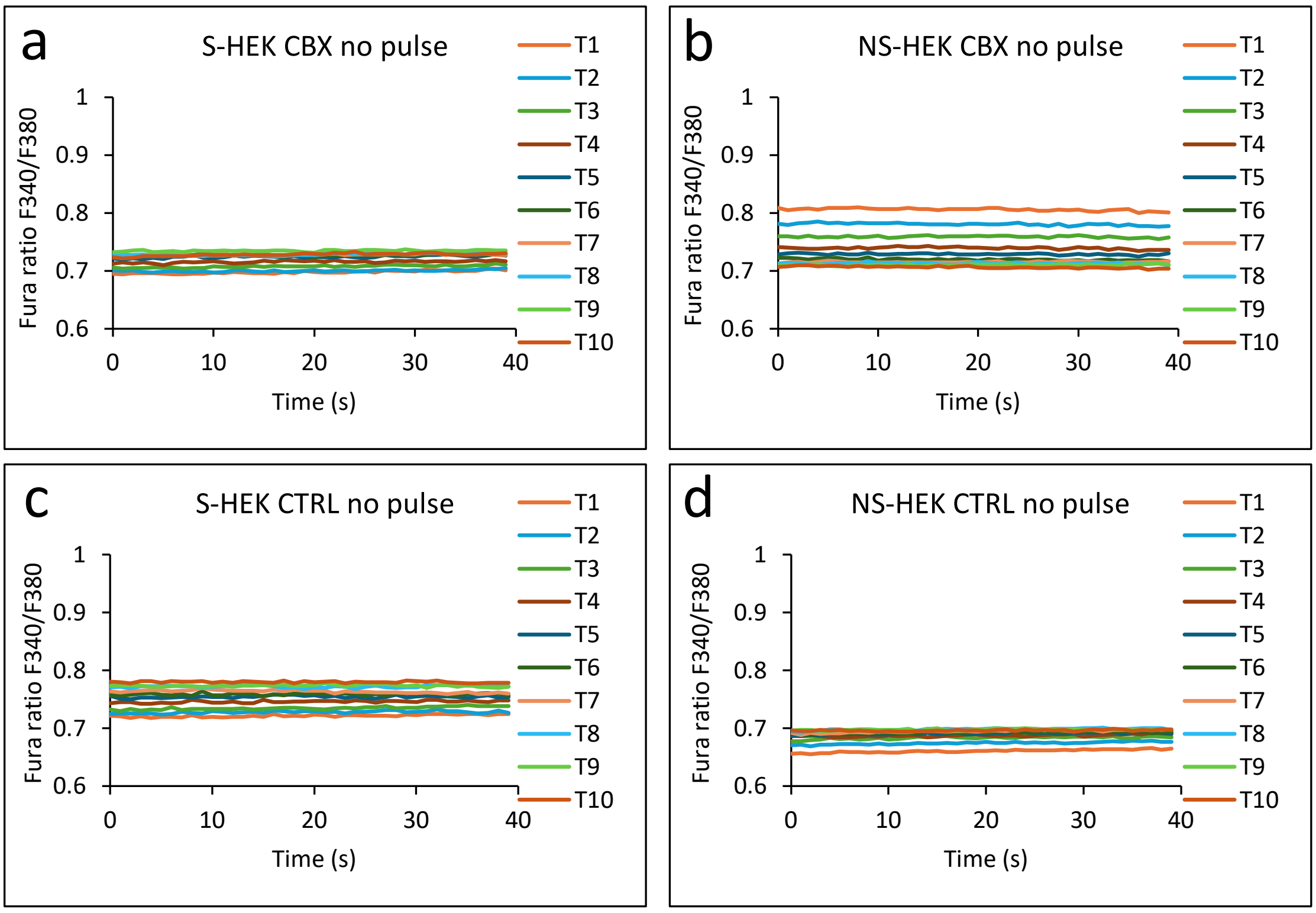

Figure S10: The effect of gap junction inhibitor carbenoxolone (CBX) on basal internal Ca concentration in S-HEK and NS-HEK cells. S-HEK (**a**) and NS-HEK cells (**b**) were incubated in 100 µM CBX and then internal Ca concentration was monitored at the same time points as before (Fig. 6) but without electric pulses. Number of experiments (N): **a**: 8, **b**: 6. For comparison, only vehicle for control were added to S-HEK (**c**) and NS-HEK cells (**d**). Ca^{2+} responses from representative experiments are shown. Image acquisition was done every two minutes (ten acquisitions completed in 20 min). Ca^{2+} responses were determined by Fura-2 ratio 340/380, averaged over the whole image. N: **c**, **d**: 4.

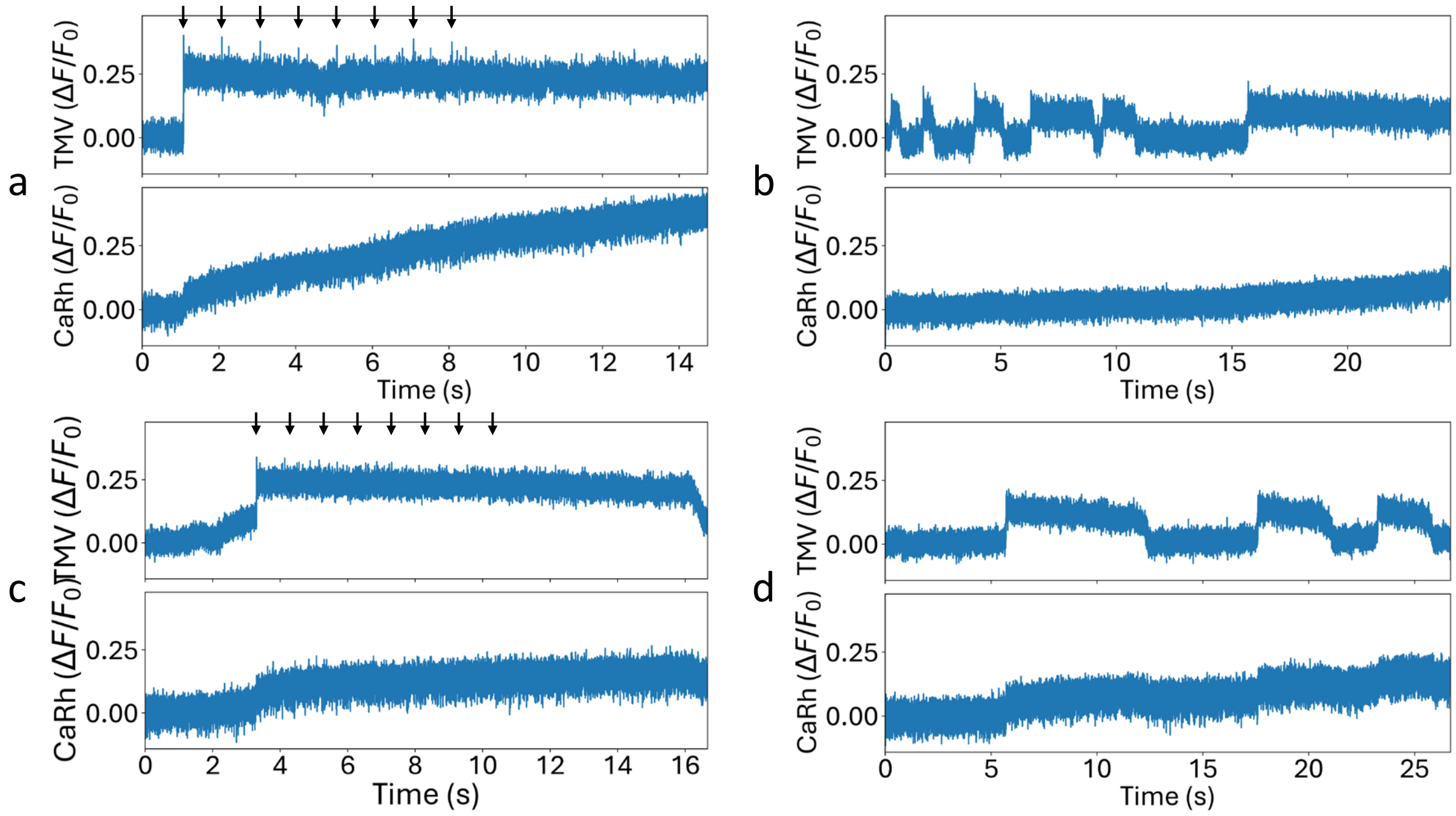

Figure S11: Examples of multiple spontaneous depolarizations, observed after pulse delivery in isolated (individual) S-HEK cells. TMV (upper figures) and intracellular Ca^{2+} (lower figures) changes were recorded simultaneously, as described in the Methods. Examples represent TMV and intracellular Ca^{2+} changes (**a**) during delivery of 8×100 µs pulses at 350 V/cm, (**b**) spontaneous depolarization in the same cell 3 min after delivery of pulses in (**a**). **c**: During delivery of 8×100 µs pulses at 250 V/cm for different isolated cell, and (**d**) spontaneous depolarizations 1 min after delivery of (**c**). Black arrows pinpoint the delivery of 100 µs pulses.

To evaluate whether electric pulses can trigger multiple APs in single S-HEK cells, we cultured S-HEK cells in monolayer for two or three days, trypsinized them and diluted them 1:5. Cells were left in culture medium for 30 min to attach to the surface of the imaging chamber.

Out of 84 observed areas with separate cells, we noticed spontaneous APs or additional APs, formed as a consequence of pulse delivery in 26 cases (30.9 %). Examples of such spontaneous APs, observed after pulse delivery can be seen in Figure S11.

It is important to note that it was often very hard to analyse the recorded data due to bad signal to noise ratios, caused by low fluorescence of small number of cells.

In few cases we also noticed that single S-HEK cells were switching between polarized and depolarized state spontaneously even in absence of any prior pulse delivery (**Figure S12**).

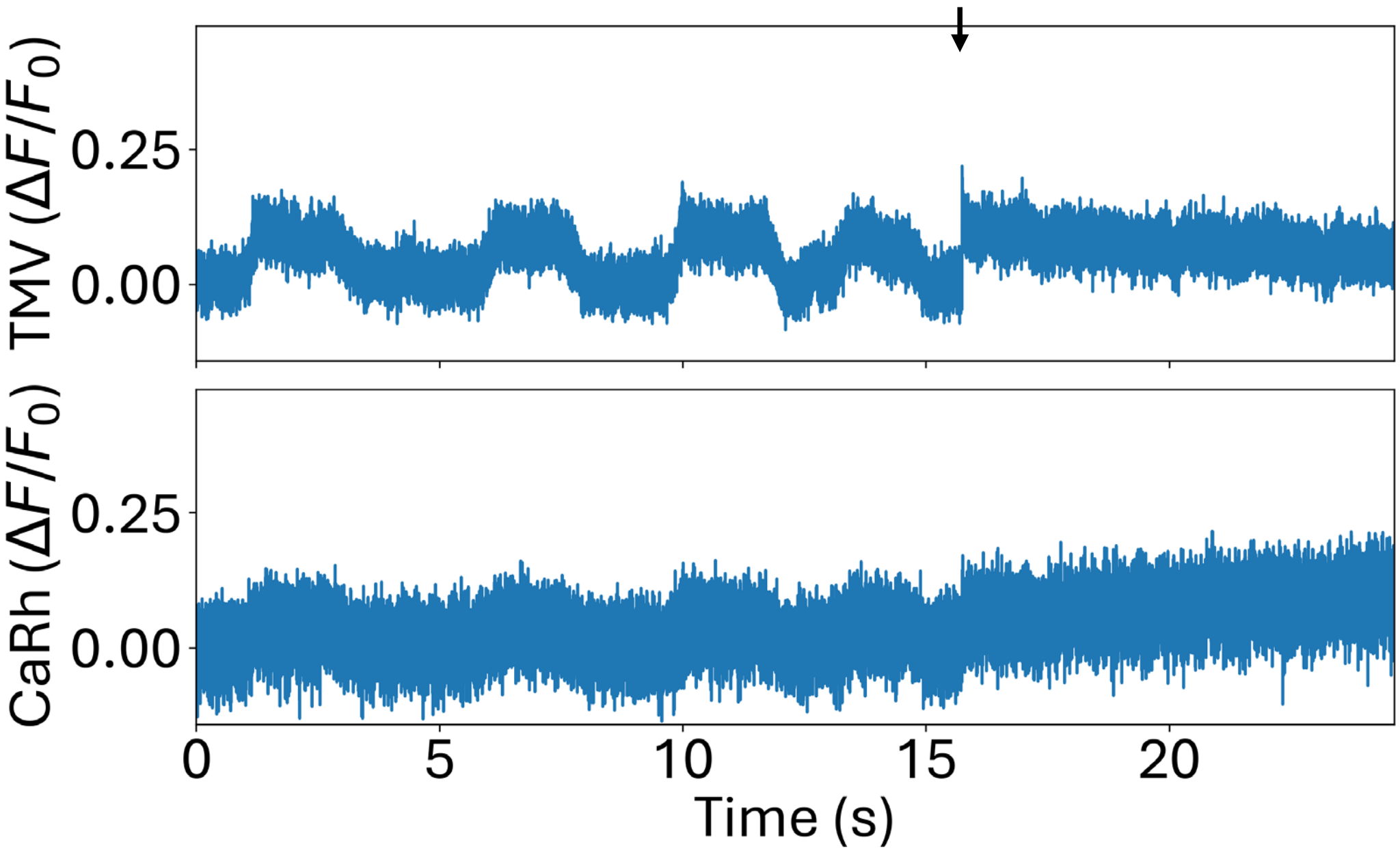

Figure S12: Example of spontaneous switching between polarized and depolarized state in single S-HEK before the delivery of a single 100 μs pulse. TMV (upper figures) and intracellular Ca^{2+} (lower figures) changes were recorded simultaneously, as described in the Methods. Example represents spontaneous TMV and intracellular Ca^{2+} changes without pulse exposure (at time 0-15 s). Black arrow pinpoints the delivery of a 100 μs pulse at 240 V/cm.

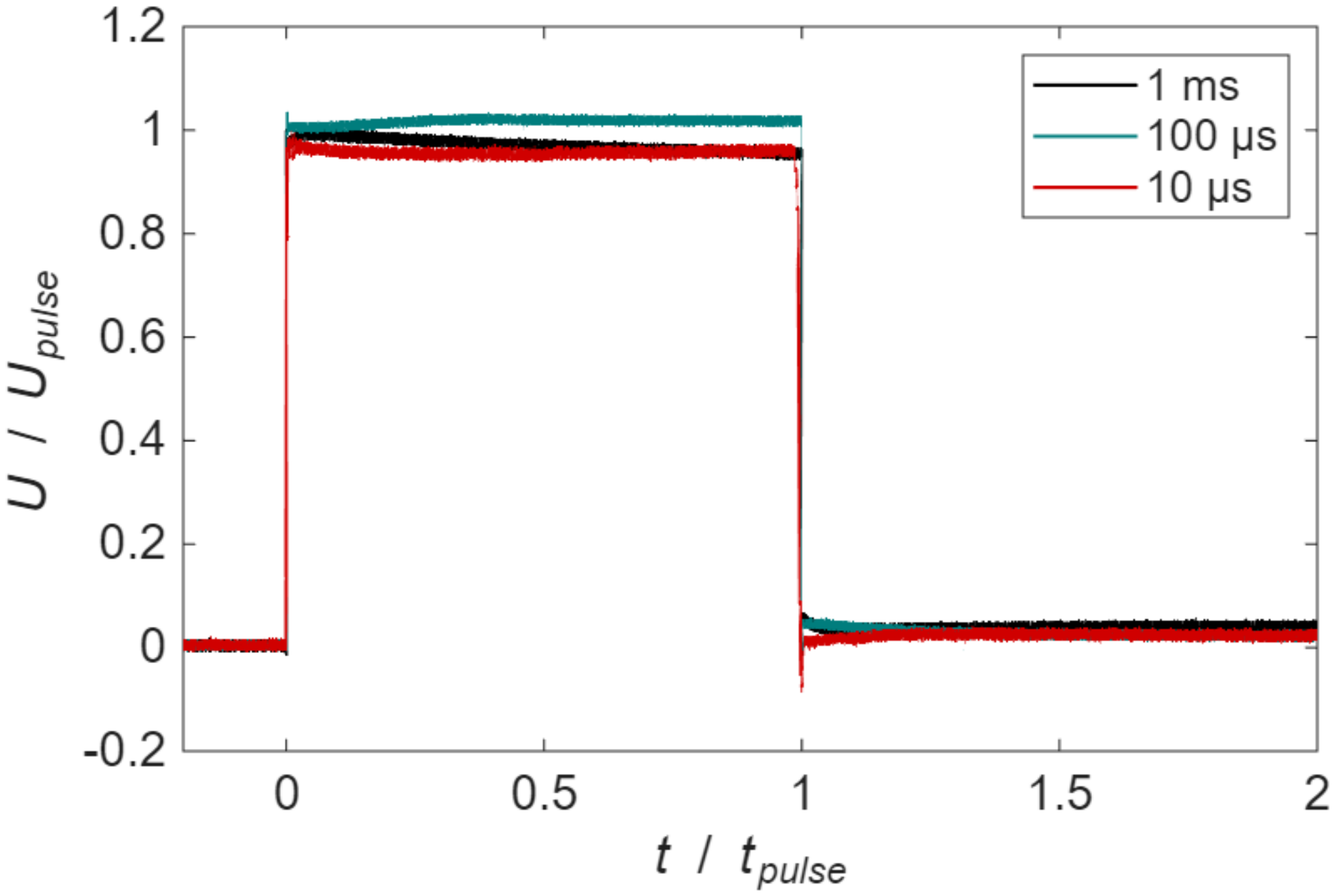

Figure S13: The shape of the pulses to which the cells were exposed. The measured voltage *U* is normalized to the voltage set on the pulse generator (U_{pulse}). U_{pulse} was 150 V for 10 µs, 88 V for 100 µs and 60 V for 1 ms electric pulse. The time *t* is normalized to the set pulse length t_0.

Table S1: Pulse amplitudes (E=U/d) used in experiments for each pulse duration, with a list of methods where these pulses were used.

Pulse duration	Electric field (V/cm)	Used in method
10 µs	240	Ca^{2+} response, simultaneous TMV/ Ca^{2+}
	280	Ca^{2+} response, simultaneous TMV/ Ca^{2+}
	300	Ca^{2+} response, simultaneous TMV/ Ca^{2+}
	330	Ca^{2+} response, simultaneous TMV/ Ca^{2+}
	360	Ca^{2+} response, simultaneous TMV/ Ca^{2+}
	400	Ca^{2+} response, simultaneous TMV/ Ca^{2+}, TMV (CBX)
	450	Ca^{2+} response, simultaneous TMV/ Ca^{2+}, TMV (CBX)
	500	Ca^{2+} response, simultaneous TMV/ Ca^{2+}
	600	Ca^{2+} response, simultaneous TMV/ Ca^{2+}
	800	Ca^{2+} response (higher E)
	1000	Ca^{2+} response (higher E)
	1200	Ca^{2+} response (higher E)
	1400	Ca^{2+} response (higher E)
	1600	Ca^{2+} response (higher E)
	1800	Ca^{2+} response (higher E)
	2000	Ca^{2+} response (higher E)
100 µs	126	TMV
	150	TMV
	176	TMV
	200	TMV
1 ms	180	Ca^{2+} response (higher E)
	230	Ca^{2+} response (higher E)
	280	Ca^{2+} response (higher E)
	330	Ca^{2+} response (higher E)
	380	Ca^{2+} response (higher E)
	430	Ca^{2+} response (higher E)
	480	Ca^{2+} response (higher E)
	530	Ca^{2+} response (higher E)
	580	Ca^{2+} response (higher E)
	800	Ca^{2+} response (higher E)

Table S2: Expression levels of calcium voltage-gated ion channels and connexins in HEK293 cells according to Human Protein Atlas on January 1, 2026. Expression was determined on mRNA level and reported in normalized transcript expression values, denoted nTPM. Proteins with the highest expression level are marked green.

Ca_V type	Channel	Gene	RNA Expression level (nTPM)
L-type	$Ca_V1.1$	CACNA1S	0.0
	$Ca_V1.2$	CACNA1C	0.8
	$Ca_V1.3$	CACNA1D	0.3
	$Ca_V1.4$	CACNA1F	0.1
P/Q-type	$Ca_V2.1$	CACNA1A	0.0
N-type	$Ca_V2.2$	CACNA1B	0.3
R-type	$Ca_V2.3$	CACNA1E	0.0
T-type	$Ca_V3.1$	CACNA1G	0.4
	$Ca_V3.2$	CACNA1H	9.8
	$Ca_V3.3$	CACNA1I	0.0
**Connexins**	**Gene**		**RNA Expression level (nTPM)**
Cx43	GJA1		48.4
Cx46	GJA3		1.8
Cx37	GJA4		0.0
Cx40	GJA5		0.2
Cx50	GJA8		0.0
Cx59	GJA9		0.0
Cx62	GJA10		0.0
Cx32	GJB1		0.0
Cx26	GJB2		1.7
Cx31	GJB3		0.0
Cx30.3	GJB4		0.0
Cx31.1	GJB5		0.0
Cx30	GJB6		0.0
Cx25	GJB7		0.1
Cx45	GJC1/GJA7		18.5
Cx47	GJC2/GJA12		0.8
Cx31.3	GJC3		0.1
Cx36	GJD2/GJA9		0.0
Cx31.9	GJD3/GJA11/GJC1		4.3
Cx40.1	GJD4		0.0
Cx23	GJE1		0.0
**HCN channels**	**Gene**		**RNA Expression level (nTPM)**
HCN1	HCN1		0
HCN2	HCN2		11.8
HCN3	HCN3		2.9
HCN4	HCN4		0
